# *Schistosoma mansoni* immunomodulatory molecule Sm16/SPO-1/SmSLP is a member of the trematode-specific helminth defence molecules (HDMs)

**DOI:** 10.1371/journal.pntd.0008470

**Published:** 2020-07-09

**Authors:** Jenna Shiels, Krystyna Cwiklinski, Raquel Alvarado, Karine Thivierge, Sophie Cotton, Bibiana Gonzales Santana, Joyce To, Sheila Donnelly, Clifford C. Taggart, Sinead Weldon, John P. Dalton

**Affiliations:** 1 School of Biological Sciences, Queen’s University Belfast, Northern Ireland; 2 Airway Innate Immunity Group (AiiR), Wellcome Wolfson Institute for Experimental Medicine (WWIEM), School of Medicine, Dentistry and Biomedical Sciences, Queen’s University Belfast, Northern Ireland; 3 Center of One Health (COH) and Ryan Institute, School of Natural Science, National University of Ireland Galway, Galway, Ireland; 4 School of Life Sciences, Faculty of Science, The University of Technology Sydney, Ultimo, NSW, Australia; 5 Institute of Parasitology, McGill University, Montreal, Quebec, Canada; University of Pennsylvania, UNITED STATES

## Abstract

**Background:**

Sm16, also known as SPO-1 and SmSLP, is a low molecular weight protein (~16kDa) secreted by the digenean trematode parasite *Schistosoma mansoni*, one of the main causative agents of human schistosomiasis. The molecule is secreted from the acetabular gland of the cercariae during skin invasion and is believed to perform an immune-suppressive function to protect the invading parasite from innate immune cell attack.

**Methodology/Principal findings:**

We show that Sm16 homologues of the Schistosomatoidea family are phylogenetically related to the helminth defence molecule (HDM) family of immunomodulatory peptides first described in *Fasciola hepatica*. Interrogation of 69 helminths genomes demonstrates that HDMs are exclusive to trematode species. Structural analyses of Sm16 shows that it consists predominantly of an amphipathic alpha-helix, much like other HDMs. In *S*. *mansoni*, Sm16 is highly expressed in the cercariae and eggs but not in adult worms, suggesting that the molecule is of importance not only during skin invasion but also in the pro-inflammatory response to eggs in the liver tissues. Recombinant Sm16 and a synthetic form, Sm16 (34–117), bind to macrophages and are internalised into the endosomal/lysosomal system. Sm16 (34–117) elicited a weak pro-inflammatory response in macrophages *in vitro* but also suppressed the production of bacterial lipopolysaccharide (LPS)-induced inflammatory cytokines. Evaluation of the transcriptome of human macrophages treated with a synthetic Sm16 (34–117) demonstrates that the peptide exerts significant immunomodulatory effects alone, as well as in the presence of LPS. Pathways most significantly influenced by Sm16 (34–117) were those involving transcription factors peroxisome proliferator-activated receptor (PPAR) and liver X receptors/retinoid X receptor (LXR/RXR) which are intricately involved in regulating the cellular metabolism of macrophages (fatty acid, cholesterol and glucose homeostasis) and are central to inflammatory responses.

**Conclusions/Significance:**

These results offer new insights into the structure and function of a well-known immunomodulatory molecule, Sm16, and places it within a wider family of trematode-specific small molecule HDM immune-modulators with immuno-biotherapeutic possibilities.

## Author summary

Sm16 is a low molecular weight protein (~16kDa) secreted by *Schistosoma mansoni*, a causative agent of human schistosomiasis. The molecule is secreted by the infectious cercariae during skin invasion and performs an immune-suppressive function to protect the invading parasite from immune attack. Using phylogenetic and gene structure analysis we show that Sm16 homologues of parasites belonging to the Schistosomatoidea superfamily of digenean worms are members of the helminth defence molecule (HDM) family that are potent immune-modulators and exclusive to trematode species. Structural analyses revealed that Sm16, much like other HDMs, consists predominantly of an amphipathic alpha-helix. Sm16 is highly expressed in the cercariae and eggs of *S*. *mansoni* but not male or female adult worms, suggesting that the molecule is of importance not only during skin invasion but also in the pro-inflammatory response to eggs in the liver tissues. A synthetic form of the molecule, termed Sm16 (34–117), was shown to bind to and enter immune cells (macrophages) and induce a weak pro-inflammatory response. However, this peptide also blocked the pro-inflammatory effects of bacterial endotoxin (lipopolysaccharide, LPS). Analysis of the transcriptome of Sm16 (34–117)-stimulated macrophages in the presence or absence of LPS suggests that it mediates immunomodulatory activity via signalling pathways that are intricately involved in regulating cellular metabolism (fatty acid, cholesterol and glucose homeostasis) and central to inflammatory responses. These new insights into the structure and function of a well-known immunomodulatory molecule, Sm16, places it within a wider family of trematode-specific small molecule HDM immune-modulators with immuno-biotherapeutic possibilities.

## Introduction

Human schistosomiasis is a public health issue affecting approximately 200 million people in over 74 tropical/sub-tropical countries, with many more people at risk of infection [1]. The causative pathogens are digenean trematode parasites of the genus *Schistosoma*, mainly *Schistosoma mansoni*, *S*. *japonicum* and *S*. *haematobium*. Chronic schistosomiasis has a significant impact on morbidity and mortality as it affects the immune system, fertility, growth, and development throughout life [2].

Schistosomiasis is acquired by contact with water containing free-swimming schistosome larvae, cercariae, that attach to and penetrate the skin. Itching or a rash on the skin can occur at the parasite’s point of entry. After a period of migration in the host, the worms mature to adults and reside as male-female pairs either in mesenteric venules (*S*. *mansoni* and *S*. *japonicum*) or in the venous plexus of the bladder (*S*. *haematobium*), where they produce approximately 300 (*S*. *mansoni*) to 3,500 (*S*. *japonicum*) eggs per day [3]. Eggs are passed through blood vessels and the wall of the digestive tract or the urinary bladder, where they are subsequently passed in faeces or urine into the environment. However, eggs can become lodged in intestinal or bladder tissue, and quite often the blood flow can displace the eggs and carry them to the liver where they become lodged in the tissue.

Typically, a weak Th1-type response is observed during the initial stages of schistosomiasis before there is a shift towards a Th2-type response concurrent with the deposition of eggs in the tissues. Schistosome eggs are highly immunogenic and release soluble antigens (SEA) that can directly modulate antigen presenting cells and promote Th2-dominant responses, an immune environment that is key to the survival of adult parasites that evade expulsion for up to 40 years [3–5]. Egg-induced granulomas consisting of a mass of cells, mainly eosinophils, Th2-type CD4^+^ T-cells, and M2 macrophages, encapsulate the eggs [6,7]. While formation of granulomas is considered a protective mechanism to prevent excessive damage to host tissue, resolution of granulomatous tissue can cause considerable tissue fibrosis, particularly in cases of repeated and chronic infections [4,8].

Sm16 is a low molecular weight protein (~16 kDa) with immunomodulatory properties that is secreted by *S*. *mansoni* cercariae as they penetrate the host skin. Sm16 expression has been described as stage-specific, with early reports indicating that it is expressed exclusively by sporocysts, cercariae, and early schistosomulae of *S*. *mansoni* [9,10]. Developmental expression analysis of *S*. *japonicum* suggested that the Sm16 homolog, Sj16, is enriched in eggs, miracidia, sporocysts, cercariae, and lung stage schistosomulae [11]. Recently, however, Bernardes et al. [12] reported that Sm16 is expressed in cercariae and newly transformed schistosomulae but not in adults or eggs.

Sm16 inhibits TLR-3 and TLR-4 signalling in human monocytes [13] and the activation of macrophages *in vitro* [14] and suppresses leukocyte accumulation when administered to mice [15–17]. Sj16 peptide can inhibit lipopolysaccharide (LPS)-induced nitric oxide production by macrophages, block macrophage phagocytic and migratory activity, and dendritic cell maturation [18–20]. It has also been reported to induce IFN-γ and IL-10 secreting CD4+ CD25+ Foxp3+ regulatory T cell (Treg) populations both *in vitro* and *in vivo* [21]. This immunomodulatory activity of Sm16/Sj16 has shown promise as an anti-inflammatory therapy by suppressing cutaneous inflammation when administered intra-dermally [17], reducing the severity of Freund’s-induced arthritis in rats [22] and protecting against inflammatory colitis in a murine dextran sodium sulphite (DSS) model [23].

We have previously described a family of immunomodulatory molecules found in medically important flatworms such as *Fasciola hepatica* which we termed helminth defence molecules (HDMs) [24]. We showed that *F*. *hepatica* HDM (FhHDM-1) exhibits potent anti-inflammatory properties; for example, it suppresses leukocyte accumulation and ameliorates inflammatory disease in pre-clinical murine models of type 1 diabetes and multiple sclerosis [25,26]. Here we describe phylogenetic, structural and functional links between Sm16 and HDM-like molecules and show that expression of these molecules is exclusive to trematode parasites. Our analysis verifies the expression of Sm16 in *S*. *mansoni* cercariae and eggs but not in adult male or female worms. We show that the C-terminal section of Sm16 is predominantly an uninterrupted amphiphilic α-helix that may allow the peptide to penetrate cells and enter the endosomal/lysosomal system of macrophages. Sm16 activates various inflammatory responses in macrophages, but also has potent inhibitory activity against LPS-induced inflammatory effects. RNA microarray and Ingenuity Pathway Analysis (IPA) predicted that several signalling pathways are affected by Sm16, most notably those involving transcription factors, peroxisome proliferator-activated receptor (PPAR) and liver X receptors/retinoid X receptor (LXR/RXR), which are involved in regulating the cellular metabolism of macrophages and central to controlling inflammatory responses. Our findings provide valuable new insights into the role of Sm16 in host-parasite interactions at key stages of the schistosome life-cycle and place it amongst the wider family of trematode-specific small molecule HDM immune-modulators that have potential in the development of novel immuno-biotherapeutics.

## Materials and methods

### Preparation of *S*. *mansoni* samples

*S*. *mansoni* cercariae and livers from infected mice were a gift from the laboratory of Dr. Paula Ribeiro, McGill University. Mature *S*. *mansoni* were recovered from the mesenteric veins of infected mice (kindly provided by the Biomedical Research Institute, Rockville, Maryland, USA). Worms were transferred into DMEM for one to two hours at 37°C until the adult male and female were separated. Males and females were conserved separately at -80°C for protein extraction or in RNA*later* (Ambion) for RNA extraction. Eggs were isolated from livers according to the procedure of Dalton et al. [27]. Infected mouse livers were also cut into small cubes and fixed in 4% paraformaldehyde in preparation for immunolocalization (see below). Serum was prepared from blood taken from mice infected with 35 cercariae at 5-, 10- and 20-weeks post-infection. Animal procedures were reviewed and approved by the Facility Animal Care Committee of McGill University and were conducted in accordance with the guidelines of the Canadian Council on Animal Care.

Proteins were extracted from cercariae, eggs, adult males and adult females with 200 μL of PBS pH 6.8 containing proteinase inhibitor cocktail (1 tablet/10ml; Roche, USA) using a pre-chilled Dounce homogenizer. Mixtures were submitted to three freeze-thaw cycles in a freezer set to maintain -20°C. Total proteins were recovered by centrifuging 30 minutes at 17,900 x g in a conventional tabletop microcentrifuge at 4°C. Protein concentrations were evaluated by Bradford assay.

### Production of Sm16 by recombinant expression and chemical synthesis

Recombinant Sm16 was produced in *Pichia pastoris* using the method previously described in Collins et al. [28]. The recombinant protein (residues 23–117, which excluded the signal sequence) was produced by fermentation at 30°C and 250–300 rpm in one litre BMGY broth buffered to pH 6.0 into 4 litre baffled flasks until an OD_600_ of 2–6 was reached. The cells were centrifuged at 3,000 x g for 10 minutes at room temperature and the induction initiated by resuspending the pellets in 200 ml BMMY broth and adding 1% filter–sterilized methanol every 24 hours for 3 days. The culture was then centrifuged at 16,000 x g for 30 minutes at RT. The pellets were discarded and Sm16 was isolated from the supernatant by Ni-NTA affinity chromatography. Recombinant *S*. *mansoni* cathepsin B1 (SmCB1) was produced in a similar manner as reported by Stack et al. [29].

A synthetic peptide corresponding to residues 34 to 117 of Sm16 (34–117) and various derivatives of this peptide were synthesised upon request by GL Biochem (Shanghai, China) and was dissolved in sterile, endotoxin-free water (Sigma Aldrich, UK) at 1 mg/ml and stored aliquoted at -80°C.

### Anti-Sm16 antibodies

Polyclonal antibodies were produced in rabbits against the peptide sequence ‘MDKYIRKEDLGMKMLDVAKILGRRIEKRMEYIAKKC’ of Sm16 by Genscript (New Jersey, USA). The cysteine was added to the C-terminus to facilitate conjugation to ovalbumin. Antibodies were lyophilized prior to shipping and were resuspended in ultrapure water before the specific anti-Sm16 peptide antibody was purified by immune-affinity chromatography. The Sm16 peptides were covalently immobilized to a beaded agarose support using the SulfoLink Immobilization kit for peptides following the manufacturer’s recommendations (Thermo Scientific, USA).

### Phylogenetic analyses

Homologous sequences were identified by TBLASTN analysis of the publically available genome databases at WormBase ParaSite (http://parasite.wormbase.org/index.html. Version: WBPS11) from 42 species of the phylum Nematoda and 27 species of phylum Platyhelminthes (S1 Table; S2 Table). BLAST analysis was based on the *F*. *hepatica* HDM sequence (CCA61804) and the *S*. *mansoni* Sm16 sequence (AAD26122), in addition to previously characterised HDM and Sm16 sequences from *Clonorchis sinensis*, *Opisthorchis viverrini* and *Schistosoma* spp (S1 Table; S2 Table). Inclusion criteria for phylogenetic analysis were based on primary sequence alignments and confirmation of an amphipathic helix by helical wheel projections (HeliQuest). Protein alignments were carried out using MAFFT using the ginsi options [30], which was hand-edited using Geneious (v11.1.5; https://www.geneious.com) resulting in a contiguous sequence block ranging from Leu^30^ to Lys^87^ (FhHDM nomenclature) containing the amphipathic region of the proteins. Phylogenetic trees were constructed with PhyML 3.0 [31] using the phylogenetic model LG +G+I, with five random starting trees and 1000 bootstrap support. The final tree figures were generated using FigTree (http://tree.bio.ed.ac.uk/software/figtree/).

### Structural analyses

The signal peptide at the N-terminus of Sm16 was identified using the SignalP 4.1 server [32]. The amino acid sequence of Sm16 was entered into the I-TASSER server (accessible via. https://zhanglab.ccmb.med.umich.edu/I-TASSER/) to obtain an *ab initio* prediction of the secondary structure. The I-TASSER server was also used to obtain a putative 3D model of secreted Sm16 [33]. The HeliQuest tool [34], was used to construct helical wheel projections. Circular dichroism (CD) spectra of recombinant Sm16 were recorded using a Jasco J-815 CD spectropolarimeter. Wavelength scans were performed between 190 and 250 nm in 10 mM Tris, 50 mM NaF buffer (pH 7.3) in both the presence and absence of trifluoroacetic aid (TFE) [30% and 60% (v/v)] with a sample concentration of 100 μg/ml. Spectra were recorded in a 1 mm quart cuvette at 20°C. Data below 190 nM for the native Sm16 sample were removed from analyses due to low signal-to-noise.

### Cell culture

The human acute monocytic leukaemia THP-1 cell line (ATCC, Manassas, USA) was routinely cultured (P2-30) in RPMI 1640 medium with ʟ-glutamine (2 mM) (Gibco, ThermoFisher Scientific, UK) supplemented with 10% (v/v) heat-inactivated foetal calf serum (FCS; Gibco, ThermoFisher Scientific, UK) and 1% (v/v) penicillin/streptomycin (PAA Laboratories GmbH, Pasching, Austria). Cells were seeded at a density of 2.5 x 10^5^ cells/well in 24 well plates and were differentiated to macrophages by incubating with 2 ml of medium with 162 nM phorbol 12-myristate 13-acetate (PMA; Sigma Aldrich, UK) for 72 hrs, then rested in fresh media (PMA-free) for 24 hrs before use. Cells were incubated with peptides (20 μg/ml) and/or LPS from *Pseudomonas aeruginosa* (100 ng/ml, Serotype 10, Source strain ATCC 27316; Sigma Aldrich, UK) in media for 16 hrs.

### Isolation and culture of bone marrow derived macrophages (BMDM)

Bone marrow was harvested from C57BL/6 and Balb/c mice and differentiated into macrophages over 6 days in RPMI medium supplemented with 10% FCS, penicillin/streptomycin (100 U/ml), _L_-Glutamine (2 mM), 2-mercaptoethanol (2-ME; 50 μM) and macrophage colony-stimulating factor (M-CSF;50 ng/ml; eBiosciences). For experimentation, cells were counted by trypan blue exclusion, seeded at a density of 1.25 x 10^5^ cells/well, and left to adhere overnight. Cells were stimulated in fresh RPMI medium with 10% FCS, penicillin/streptomycin (100 U/ml), and _L_-Glutamine (2 mM) for 24 hrs. Cell-free supernatants were collected for measurement of cytokines (stored at -20°C until required). For dose-dependency response studies, Balb/c bone marrow derived macrophages (5.0 x 10^5^) were incubated for 30 min with full-length Sm16 (34–117) (5–50 μg/ml) and after washing in PBS were then stimulated with LPS (10 ng/ml) overnight. Ethical approval for these studies was granted by the University of Technology Sydney (UTS) Animal Care and Ethics Committee (Approval Number: 2017–1232) and experiments were conducted in accordance with the approved guidelines to be compliant with The Australian Code for the Care and Use of Animals for Scientific Purposes.

### RNA extraction, cDNA synthesis and qPCR

Total RNA was extracted from adult males, adult females, mixed adults, eggs and cercariae using the miRNeasy Mini Kit (Qiagen, UK) according to the manufacturer’s instructions, eluted in 30 μl RNase-free water. Assessment of RNA concentration and quality was carried out using the LVis plate functionality on the PolarStar Omega Spectrophotometer (BMG LabTech, UK). cDNA synthesis was carried out using the High capacity cDNA reverse transcription kit (ThermoFisher Scientific, UK) according to manufacturer’s instructions.

Quantitative PCR (qPCR) reactions were performed in 20 μl reaction volumes in triplicate, using 1 μl cDNA, 10 μl of Platinum SYBR Green qPCR SuperMix-UDG kit (ThermoFisher Scientific, UK) and 1 μM of each primer to amplify the Sm16 gene (Sm16_F 5’-CCGAGTGAAAAAGACATGGAAT-3’ and Sm16_R 5’-TCAATGCGTCTTCCAAGGAT-3’), and the constitutively expressed *S*. *mansoni* PAI gene (SmPAI_F 5’-ACGACCTCGACCAAACATTC-3’ and SmPAI_R 5’-TAGCTCCGACAGAAGCACCT-3’). qPCR was performed using a Rotor-Gene thermocycler (Qiagen, UK), with the following cycling conditions: 95°C for 10 s, 50°C for 15 s and 72°C for 20 s. Relative expression analysis was performed manually using Pfaffl's Augmented ΔΔCt method [35], whereby the comparative cycle threshold (Ct) values of the samples of interest are compared to a control and normalised to the PAI gene expression. The data are presented relative to the level of Sm16 expression in male adult schistosomes. Results were analysed using One Way ANOVA (version 6.00 for Windows, GraphPad Software); P-value <0.05 was deemed statistically significant.

### Immunolocalization in *S*. *mansoni* eggs

Paraformaldehyde-fix liver sections were put into embedding cassettes and were dehydrated in sequential ethanol baths ranging from 50 to 100% with the last two steps in xylene substitute. Then, tissues were infiltrated with paraffin wax and blocks were placed on a cooling plate for 15 min to solidify. Five μm sections, cut using a microtome, were floated in a 45°C water bath and put on slides. Slides were allowed to dry at RT overnight before the immunolocalization procedure.

For immunolocalization, slides were put in Safeclear (Xylene substitute; ThermoFisher Scientific, USA) three times for two minutes. They were subsequently rehydrated by sequential dipping in ethanol ranging from 100% to 20% with a final step in water. Sections were treated for two hours at RT in 2% BSA-PBS. They were then incubated overnight at 4°C with rabbit anti-Sm16 (1:100). After three washes of five minutes in PBS, tissues were incubated for 1 hour with the Alexa Fluor 488-conjugated anti-rabbit (Invitrogen, USA; 1:1000) in 2% BSA-PBS at RT and protected from light. After a wash of five minute in PBS, DAPI (dilactate; Invitrogen, USA; 1:750 in PBS) was added and incubated for five minutes at RT. Tissues were washed three times for five minutes with PBS and mounted with PERMOUNT with a drop of mounting media. Confocal microscopy was performed with a BIO-RAD RADIANCE 2100 confocal laser scanning microscope (CLSM) equipped with a Nikon E800 fluorescence microscope for confocal image acquisition and the LASERSHARP 2000 software package.

### Internalisation of Sm16 by BMDMs

BMDMs (7 x 10^6^) were treated with 10 μg/ml of Alexa Fluor 488-conjugated (Life Technologies, Vic Australia) recombinant Sm16 or peptide Sm16 (34–117) for 30 min at 37ºC then washed and fixed with 4% paraformaldehyde and permeabilized with 0.1% Triton-X/PBS. Samples were also stained with DAPI for identification of the cell nucleus. To follow internalisation of Sm16 (34–117), BMDMs (7x10^6^) were simultaneously incubated with 10 μg/ml of Alexa Fluor 488-labelled Sm16 (34–117) and 60 nM LysoTracker (Life Technologies, Vic, Australia) and imaged live after 30 min at 37ºC as described by Robinson et al. [36].

### Immunoblot with infected mouse sera

To analyse the proteins by immunoblotting they were first resolved by 12% SDS-PAGE. Proteins were transferred to nitrocellulose using a semi-dry blotting apparatus. The nitrocellulose membrane was blocked for 1hr at RT with 15 ml of 5% milk in TBS/0.05% Tween-20. Then, 15ml of 2.5% milk in TBS/0.05% Tween-20 containing the primary antibody (anti-Sm16 or serum from infected mice) was added to the nitrocellulose membrane for 1 hr, with rotation at RT. The nitrocellulose was washed three times for five min each with TBS/0.05% Tween-20 and then incubated in 15 ml of secondary antibody-peroxidase conjugate in TBS/0.05%-Tween for 1hr at RT. The nitrocellulose was washed three times for five min each with TBS/0.05 Tween-20 and then incubated in 15 ml of secondary antibody-peroxidase conjugate in TBS/0.05%-Tween for 1 hr at RT. The nitrocellulose filter was again washed three times for five min each. Bound antibody was visualized by adding 1 ml of each reagent of SuperSignal West Femto Chemiluminescence Substrate (ThermoFisher Scientific, USA) for 5 minutes. The membrane was dried and developed in the dark using the autoradiography cassette and Kodak X-OMAT 2000 processor system.

### Cytokine analysis

Human cytokines were measured using human IL-6 uncoated ELISA kit (Invitrogen, ThermoFisher Scientific, UK), human TNF standard ABTS ELISA kit (Peprotech, London, UK), and human IL-8 ELISA MAX standard kit (Biolegend, San Diego, CA, USA) according to the manufacturers’ instructions. Cytokine arrays used were Human Cytokine Array C3 (RayBiotech, Norcross, GA, USA). The levels of mouse cytokines present in culture supernatants were quantified using an ELISA (BD Pharmingen, North Ryde, NSW, Australia), according to the manufacturer’s instructions.

### RNA microarrays

Cells obtained from three independently performed experiments were lysed in 400 μl TRIzol Reagent (ThermoFisher Scientific, UK) and RNA was purified using PureLink RNA Mini Kit (ThermoFisher Scientific, UK). RNA integrity number (RIN) scores were determined using RNA 6000 nano gel matrices (Agilent Technologies, Santa Clara, CA, USA). Microarray analysis of RNA (100 ng/μl; RIN score ≥ 9.9) was carried out using Human HT-12 v4 BeadChips (Illumina, San Diego, CA, USA). Differential gene expression analysis was carried out using Partek Genomics Suite (PGS) version 6.6 (Partek Incorporated, Chesterfield, MO, USA). Genes were filtered for fold change in > 1.5 and < -1.5 and an expression p-value <0.05. False discovery rate (FDR) correction was not applied. The canonical pathway and comparison analyses were generated through the use of Ingenuity Pathway Analysis (IPA) (QIAGEN Inc., https://www.qiagenbioinformatics.com/products/ingenuity-pathway-analysis).

### Statistical analysis

Results were analysed using one-way ANOVAs with Tukey’s multiple comparison test. Differences were not deemed significant when p-values (p) >0.05. *p <0.05, **p <0.01, ***p <0.001, ****p <0.0001.

## Results

### Schistosome Sm16 is a helminth defence molecule (HDM)

Analysis of genomic data available on WormBase ParaSite facilitated the identification of a number of homologues of Sm16 and HDMs. Most notably, these molecules were identified solely in the genomes of trematode species (i.e. no HDMs were discovered in the genomes of any cestode or nematode). Phylogenetic analysis of the HDM sequences recovered from the various trematode genomes demonstrates a very close relationship between Sm16-like molecules and *Fasciola-*like HDMs. However, Sm16-like molecules form a distinct branch and are exclusively produced by organisms of the Schistosomatoidea superfamily, some of which, for example *S*. *japonicum*, express several members. We have termed these the Sm16-like HDMs.

**Fig 1:**
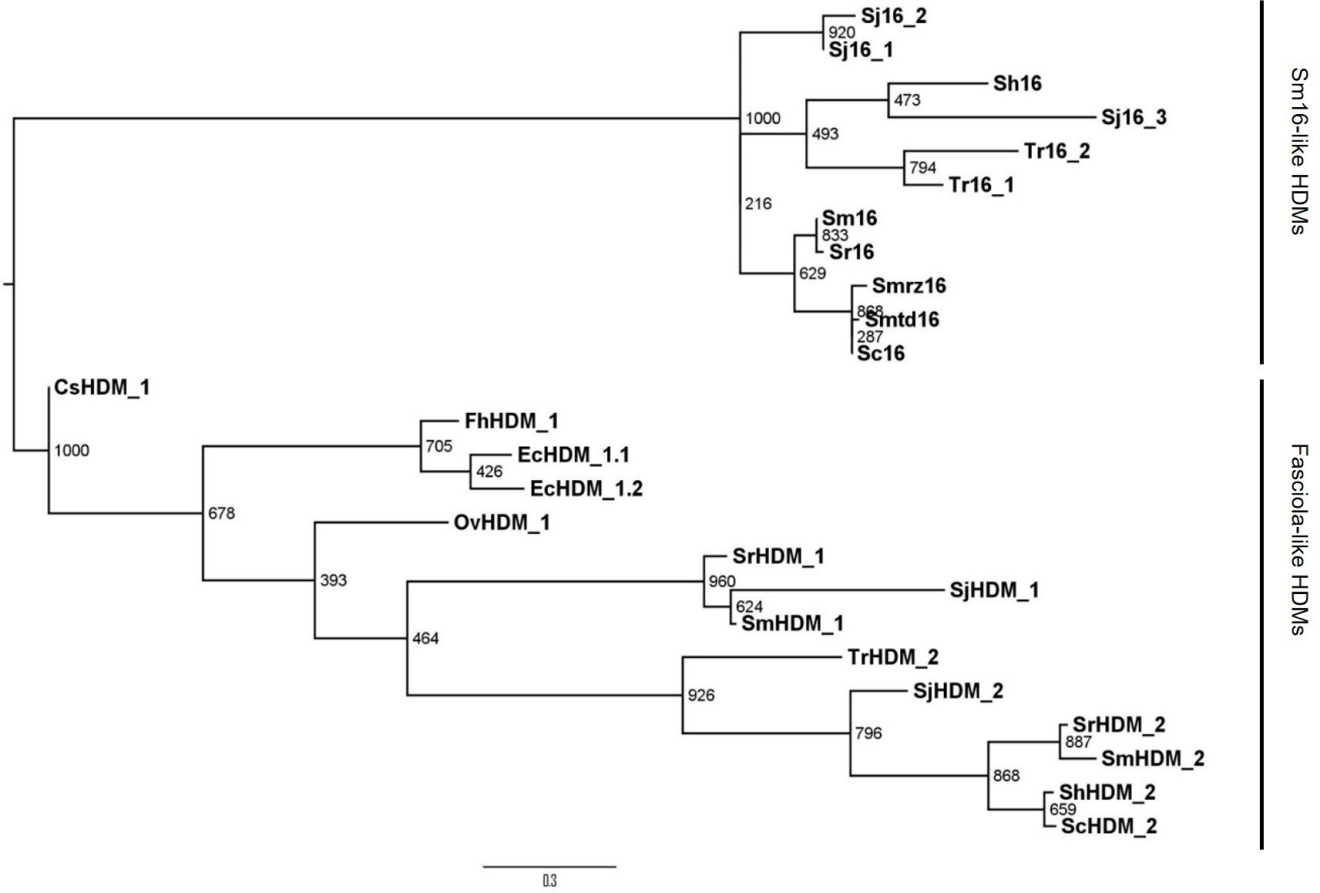
HDMs are a trematode-specific family of immunomodulatory peptides inclusive of Sm16-like molecules. Midpoint-rooted maximum likelihood phylogram of the trematode-specific HDM family generated by PhyML, based on the protein sequence Leu^30^ to Lys^87^ (FhHDM nomenclature) containing the amphipathic region of the proteins from 12 trematode species: *Clonorchis sinensis* (CsHDM_1), *Echinostoma caproni* (EcHDM_1.1 & EcHDM_1.2), *Fasciola hepatica* (FhHDM_1), *Opisthorchis viverrini* (OvHDM_1), *Schistosoma curassoni* (Sc16 & ScHDM_2), *S*. *haematobium* (Sh16 & ShHDM_2), *S*. *japonicum* (Sj16_1, Sj16_2, Sj16_3, SjHDM_1 & SjHDM_2), *S*. *mansoni* (Sm16, SmHDM_1 & SmHDM_2), *S*. *margrebowiei* (Smrz16), *S*. *mattheei* (Smtd16), *S*. *rodhaini* (Sr16, SrHDM_1 & SrHDM_2) and *Trichobilharzia regenti* (Tr16_1, Tr16_2 & TrHDM_2). The clusters of Sm16-like HDMs and *Fasciola*-like HDMs are shown. Bootstrap support values (1000 iterations) are shown at each node. Accession number/gene identifiers are presented in S1 Table.

The *Fasciola*-like HDM branch of the phylogenetic tree (Fig 1) currently contains HDMs from *F*. *hepatica*, *Echinostoma caproni*, *Clonorchis sinensis* and *Opisthorchis vivverrini* which cluster together. It also contains HDMs from various species of the Schistosomatoidea superfamily; however, these form a separate extended branch. We have termed these the *Fasciola*-like HDMs.

The evolutionary relationship between members within the Sm16-like HDMs and *Fasciola*-like HDMs is also supported by their genomic organization; the structure of the genes from both groups feature four exons separated by three introns of similar lengths. The first exon encodes the secretory signal peptide. There is a particularly high degree of sequence conservation in the third and fourth exons across all of the gene sequences that encodes the C-terminal region of the protein which is comprised mainly of α-helix secondary structure (S1 Fig, S2 Fig; discussed below).

### Structural analysis of Sm16 reveals an amphipathic α-helical molecule

**Fig 2:**
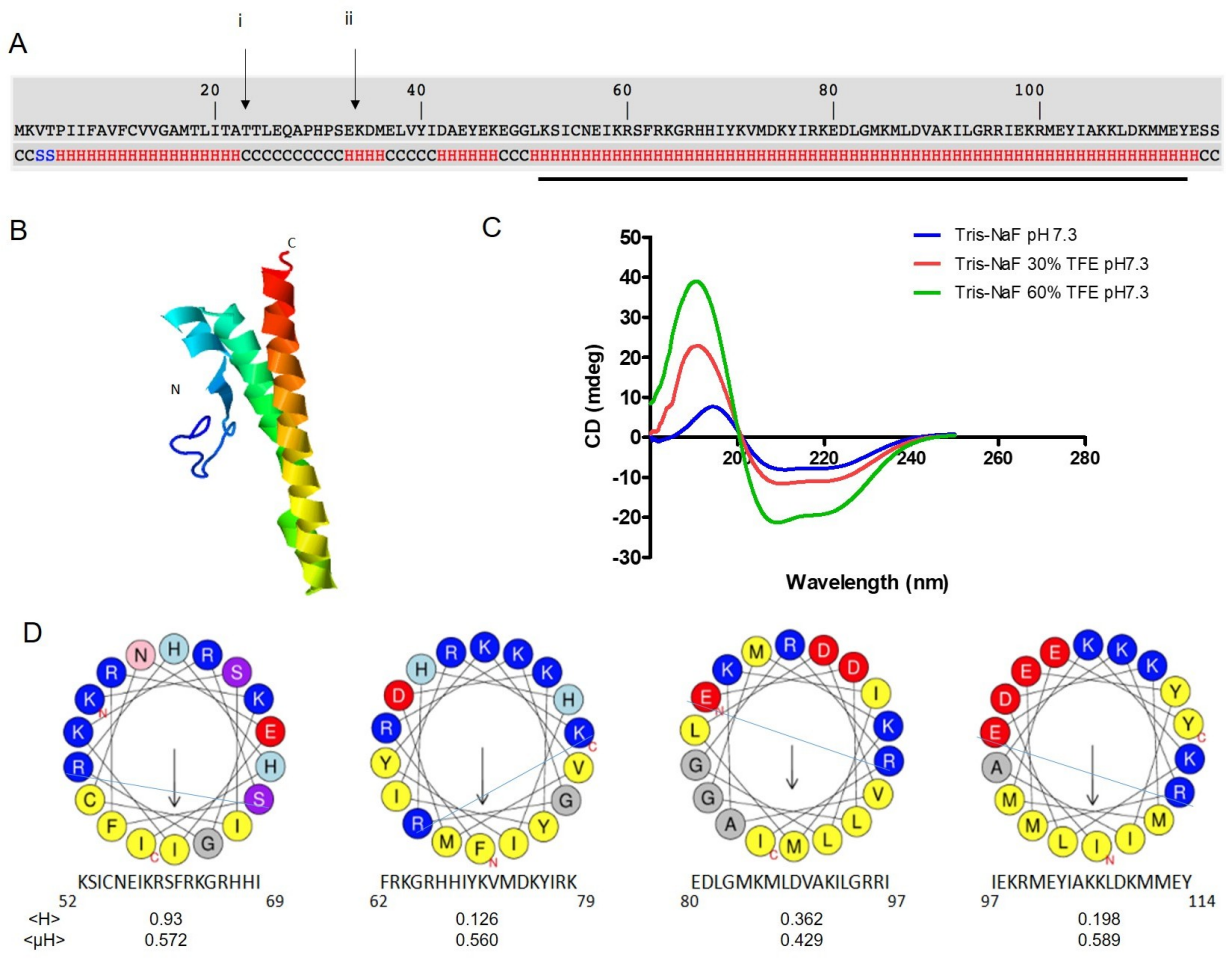
Sm16 predominantly consists of an amphipathic alpha helix. (A) Predictive secondary structure of Sm16 generated using I-TASSER: H–Helix; S–Strand; C–coil. Arrow (i) denotes the SignalP 4.1 predicted cleavage site for an N-terminal secretory signal peptide between residues 22 and 23, and arrow (ii) shows the commencement of the synthetic Sm16 (34–117) peptide sequence. The black line indicates the portion of Sm16 that is amphipathic. (B) 3D model of full length secreted Sm16, generated using I-TASSER (C) Circular dichroism analysis performed on recombinant Sm16 in the absence of tetrafluoroethylene (TFE), in 30% TFE, and 60% TFE. (D) Helical wheel analysis of Sm16 performed using HeliQuest identified four hydrophobic faces (indicated by blue line through helix) in continuous succession in the amphipathic C-terminal helix. <H>—Hydrophobicity; <μH>—Hydrophobic Moment.

Analysis of the amino acid sequence of Sm16 using I-TASSER indicated that much of the molecule is helical in structure (Fig 2A and 2B). This was further confirmed by circular dichroism analysis of the recombinant Sm16 produced in *Pichia pastoris* (Fig 2C; S3 Fig). Further analysis of the sequence using helical wheel projections (HeliQuest) indicated that the C-terminal half of Sm16 (residues 52–114) is predominantly uninterrupted amphiphilic α-helix containing four hydrophobic hotspots (Fig 2D). As mentioned above, this C-terminal section of the protein is highly conserved between the Sm16-like molecules of the Schistosomatoidea and is encoded by the third and fourth exons of their genes (S1 Fig).

### Sm16 is expressed predominantly in *S*. *mansoni* cercariae and eggs

**Fig 3:**
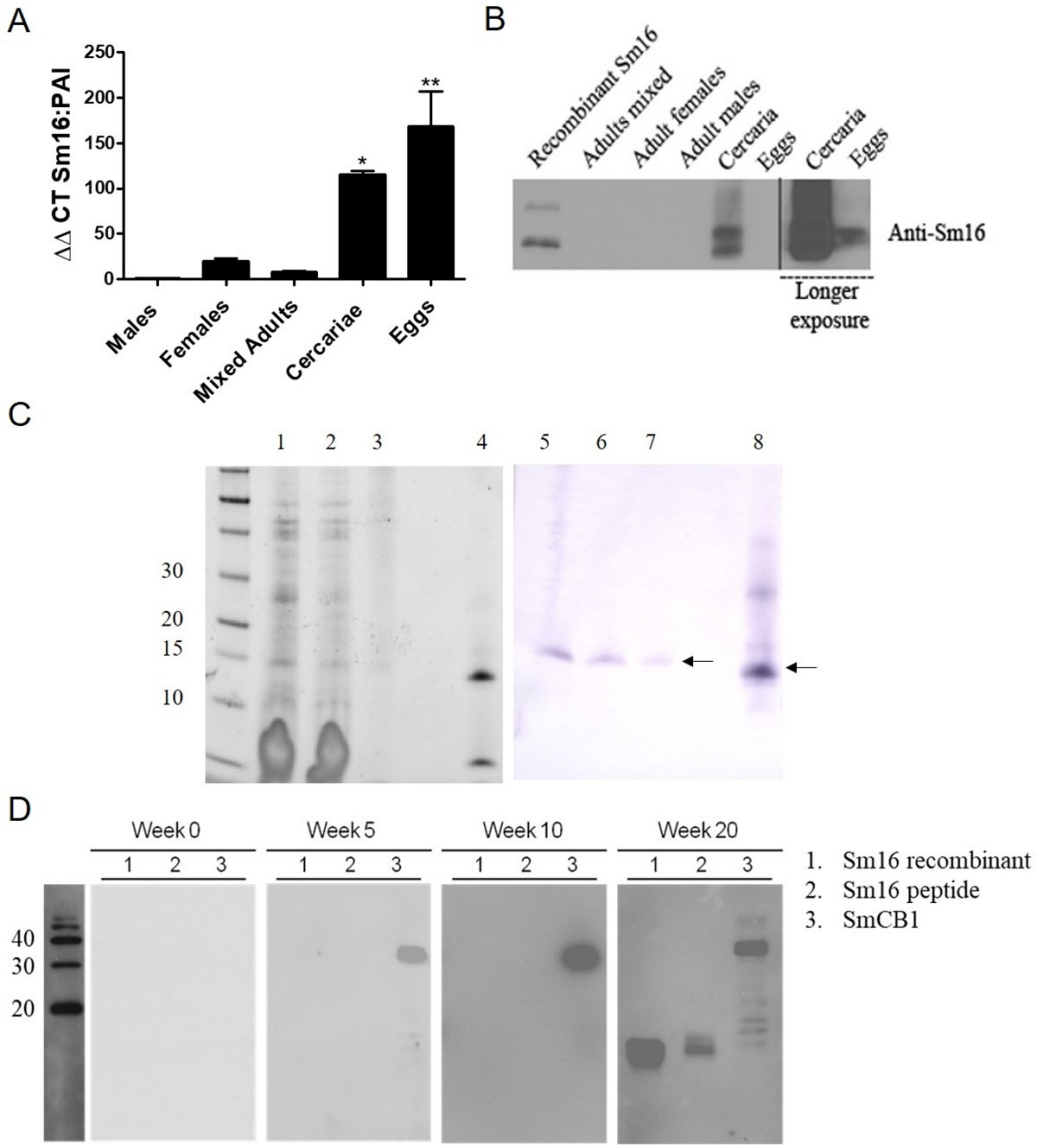
Sm16 is expressed in *S*. *mansoni* cercariae and eggs. (A) qPCR was used to assess the expression of Sm16 mRNA in *S*. *mansoni* adults (males, females, and mixed), cercariae and eggs. ΔΔCt values were normalised to the level of PAI expression in samples (3) and presented as relative to the level of Sm16 expression in male adult schistosomes and analysed by ANOVA with Tukey’s multiple comparison test. *p <0.05, **p <0.01. (B) Western blot carried out using 5 μg of crude extract from *S*. *mansoni* adults (mixed, females and males) cercariae and eggs and probed with an anti-Sm16 antibody. (C) SDS-PAGE analysis (lanes 1–4) and Western blot (5–8) of soluble extracts of cercariae (1 and 5), newly-transformed schistosomula (2 and 6), concentrated transformation medium (3 and 7) and recombinant Sm16 (4 and 8) (D) Blood samples were taken from mice with experimental schistosomiasis at 0, 5, 10, and 20 weeks post infection and sera was used to probe Western blots of recombinant Sm16, synthetic peptide Sm16 (34–117), and recombinant *S*. *mansoni* cathepsin B1 (SmCB1) (1 μg of each).

To investigate Sm16 expression in the stages of *S*. *mansoni* that exist in the mammalian host, qPCR was performed on mRNA extracted from adults (males, females, and both male and female mixed), cercariae, and eggs. Sm16 transcription was significantly higher in both *S*. *mansoni* cercariae and egg samples compared to adult male worms. Low levels of Sm16 expression were observed within the female worms and while higher than in males these levels were not statistically different (Fig 3A). Anti-Sm16 antibodies, raised against a synthetic peptide derived from Sm16 (residues 34–117) was used to probe a Western blot containing *S*. *mansoni* adults (mixed, males, and females), cercariae, and egg crude extracts. Sm16 was not detected in the adult worm samples, consistent with the data derived from qPCR (Fig 3B). Sm16 was most abundant in cercariae and was detected in eggs when the immunoblots were exposed for longer periods (see Fig 3B). Cercariae mechanically-transformed into schistosomules along with the concentrated transformation medium were also probed with anti-Sm16 antibodies. This analysis identified Sm16 in both parasite stages and in the medium demonstrating that Sm16 is released from the cercariae during the transformation process (Fig 3C). It is worth noting that mature Sm16 has a lower molecular weight with reports calculating the mature secreted protein to be between 11.3–11.7 kDa in size [10,18], but it can run slightly higher on SDS-PAGE.

### Sm16 is immunogenic in *S*. *mansoni-*infected mice, but only late in infection

In order to determine if Sm16 is immunogenic during infection, mice were experimentally infected with 35 *S*. *mansoni* cercariae and serum samples harvested at 0-, 5-, 10- and 20-weeks post-infection were used to probe Western blots containing recombinant Sm16 and synthetic Sm16 (34–117). Recombinant *S*. *mansoni* cathepsin B1 (SmCB1), an immunogenic protease that is produced and secreted abundantly by intra-mammalian *S*. *mansoni* [37] was used as a positive control. The immunoblots showed that circulating antibodies to SmCB1 are present as early as week five post-infection and remain high at week 10 and 20 post-infection. However, neither recombinant nor synthetic Sm16 preparations were detected on blots that were probed with serum obtained from mice at 5 and 10 weeks after infection but were strongly reactive with serum taken at 20 weeks post-infection (Fig 3D).

### Sm16 is detected in eggs in *S*. *mansoni-*infected mice

**Fig 4:**
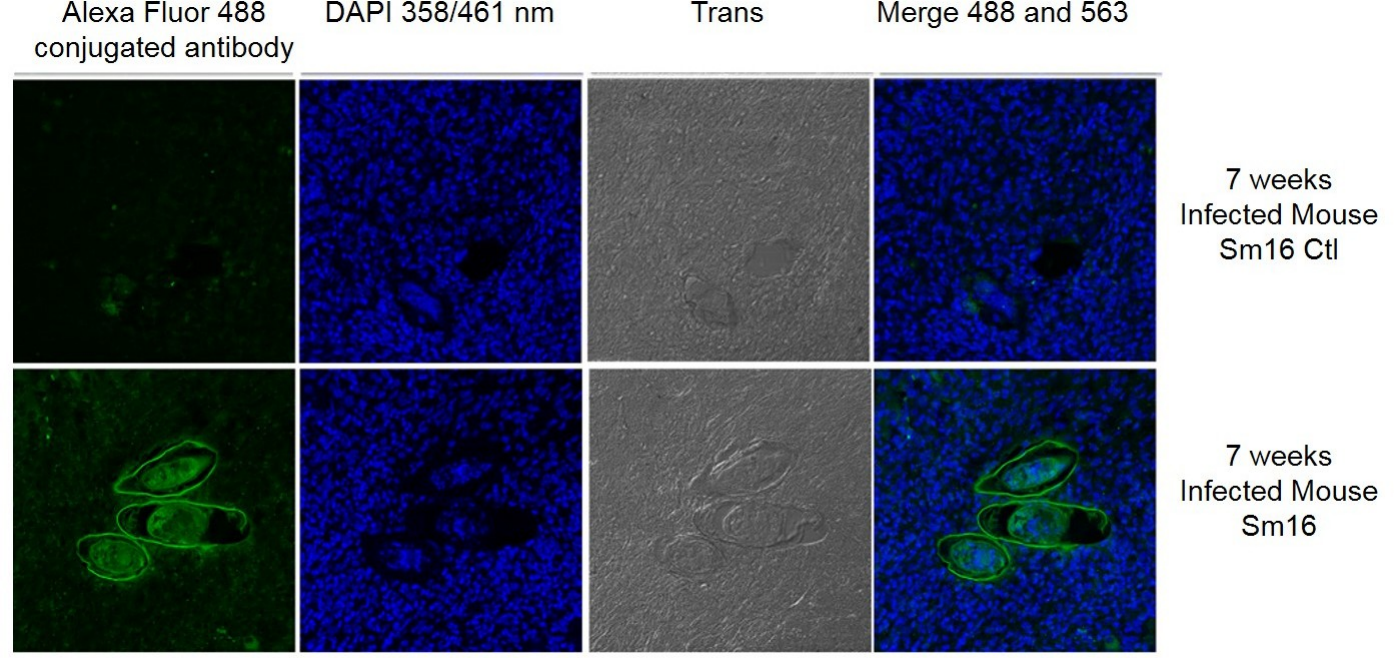
Immunolocalisation of Sm16 in *S*. *mansoni* eggs. Paraffin was embedded liver sections of *S*. *mansoni*-infected (7 weeks post-infection) containing *S*. *mansoni* eggs were probed with anti-Sm16 antibody represented by green fluorescence. For the negative controls (Ctl) the anti-Sm16 antibodies were first adsorbed with an excess of recombinant Sm16 prior to being used in the protocol. Nuclear staining was carried out using DAPI represented by blue fluorescence. The Trans panels shows the sections under light microscopy.

*S*. *mansoni* eggs were identified in sections of liver tissue from mice that had been experimentally infected with *S*. *mansoni* for seven weeks (Fig 4). Immunofluorescent imaging by means of probing with anti-Sm16 antibody followed by Alexa Fluor 488-conjugated anti-rabbit antibodies was used to confirm the presence of Sm16 in *S*. *mansoni* eggs. Anti-Sm16 antibody binding was clearly observed within the unembryonated miracidium in the eggs. No labelling was observed within eggs using control mouse serum.

### Sm16 (34–117) is taken up by macrophages and co-localises with the endo-lysosomes

**Fig 5:**
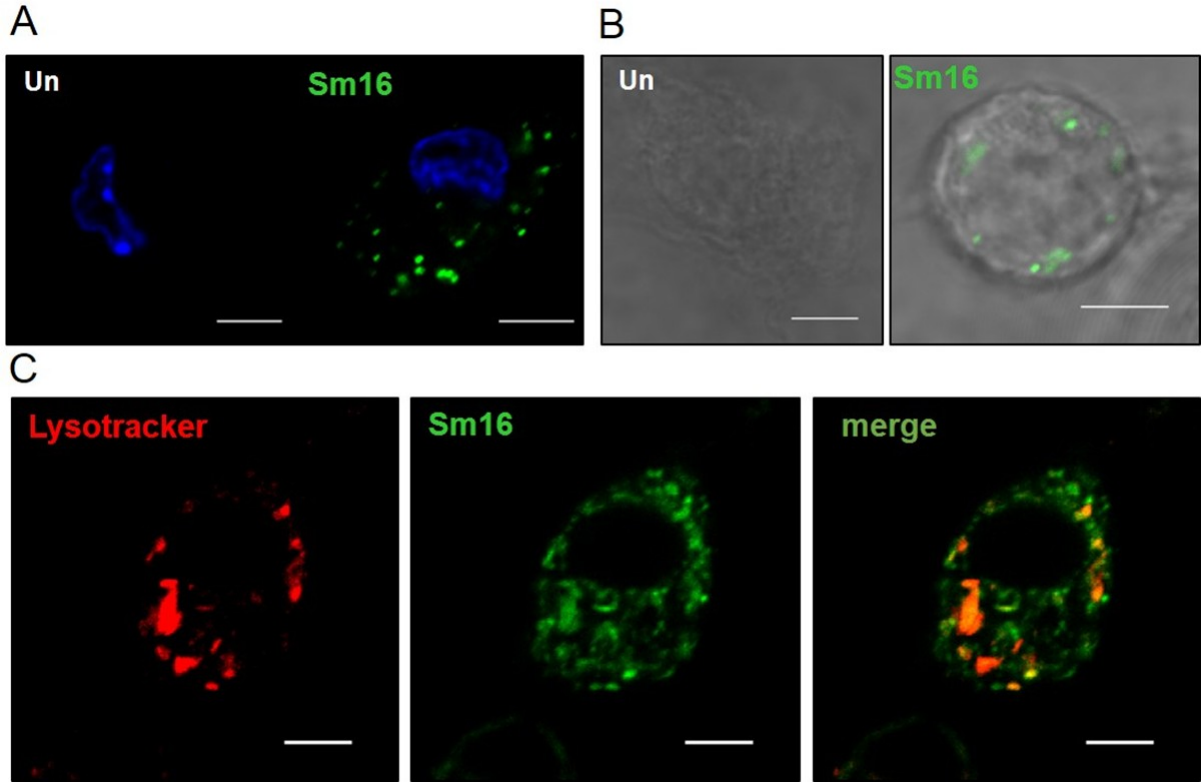
Sm16 is internalised by macrophages. (**A**) BMDMs (7 x 10^6^) were untreated (Un) or incubated with 10 μg/ml Alexa Fluor 488-conjugated recombinant Sm16 (Sm16) in media for 30 min at 37°C, 5% CO_2_, prior to fixation with 4% PFA. Samples were also stained with DAPI for identification of the cell nucleus. (**B**) BMDMs (7 x 10^6^) were untreated (Un) or incubated with 10 μg/ml Alexa Fluor 488-conjugated synthetic Sm16 (34–117) (Sm16) in media for 30 min at 37°C, 5% CO_2_, prior to fixation with 4% PFA. (**C**) BMDMs (7x10^6^) were incubated with Alexa 488-conjugated recombinant Sm16 (10μg/mL) in media with 60 nM LysoTracker for 30 min at 37°C, 5% CO2. Visual identification of fluorescence in the respective channels was used to construct the panels; Sm16 staining shown in green, DAPI staining in blue and LysoTracker staining shown in red. Co-localization identification was confirmed by automated analysis using the NIS software. Scale bar: 5μM.

The *Fasciola*-like HDMs are known to mediate at least a part of their immune modulatory effect through interaction with macrophages. To determine whether the Sm16 peptides had the same potential, we visualised the uptake of Alexa Fluor 488-conjugated recombinant Sm16 and synthetic Sm16 (34–117) by murine macrophages (Fig 5A & 5B, respectively). Both recombinant and peptide were clearly internalised by the macrophages, presented as punctate fluorescence in the cytoplasm fifteen minutes after their addition to cells in culture. Furthermore, the co-localisation of Sm16 (34–117)-conjugated fluorescence with Lysotracker indicated that the peptides were located within the endo-lysosomal system of macrophages (Fig 5C). The labelling with anti-Sm16 appeared more extensive within the endosomal/lysosomal system than Lysotracker since the latter only fluoresces within the more acidic mature lysosomes.

### Sm16 (34–117) affects cytokine production by macrophages

To evaluate the effects of Sm16 (34–117) on the inflammatory responses of human macrophages, we analyzed supernatants of THP-1 macrophages treated with Sm16 (34–117) and LPS using a broad cytokine array (S3 Table). The data showed that Sm16 (34–117) alone increased secretion of cytokines including IL-6, IL-1β, GM-CSF, I-309, TNF, and IL-10. Stimulation with LPS alone also increased secretion of these cytokines; however, the quantities of IL-6, GM-CSF, TNF were higher than in the macrophages stimulated by Sm16 (34–117) while induction of IL-1β and I-309 were lower and IL-10 the same. Therefore, both Sm16 and LPS induce a pro-inflammatory response from THP-1 macrophages, albeit with some differences. However, addition of Sm16 (34–117) to THP-1 cells alongside LPS suppressed the induction of the LPS-induced inflammatory response in macrophages (S3 Table).

**Fig 6:**
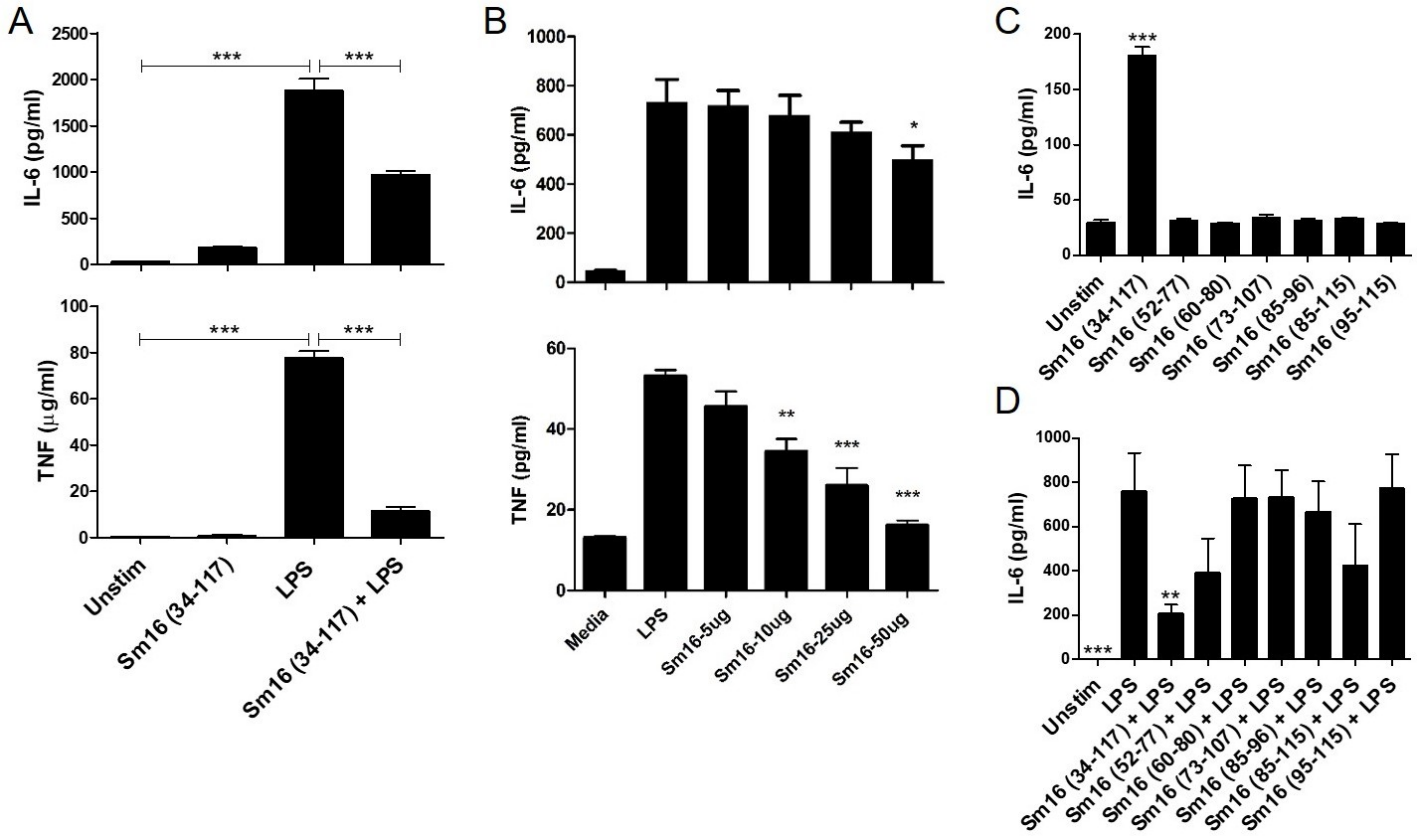
Effects of synthetic peptide Sm16 (34–117) treatment on cytokine secretion by macrophages. (**A**) IL-6 and TNF in cell supernatants of THP-1 macrophages (2.5 x 10^5^) treated with Sm16 (34–117) (20 μg/ml), LPS (100 ng/ml) and Sm16 (34–117) + LPS for 16 hrs were quantified by ELISA. Data derived from three independently performed experiments was analysed using repeated measures ANOVA with Tukey’s multiple comparison test. (**B**) Sm16 (34–117) inhibits macrophage activation in a dose-dependent manner. Bone marrow derived macrophages (5.0 x 10^5^) from Balb/c mice were incubated for 30 min with full-length Sm16 (34–117) (5–50 μg/ml) and after washing in PBS were then stimulated with LPS (10 ng/ml) 16h. TNF and IL6 in cell supernatants was measured by ELISA. (**C-D**) Effect of Sm16 (34–117) and small peptides derivatives from the C-terminal amphipathic helix. THP-1 cells were treated with 20 μg/ml of Sm16 (52–77), Sm16 (60–80), Sm16 (73–107), Sm16 (85–96), Sm16 (85–115), or Sm16 (95–115) in the absence (C) and presence (D) of LPS stimulation (100 ng/ml). IL6 in cell supernatants was measured by ELISA. Data analysed by ANOVA with Tukey’s multiple comparison test as above. *p <0.05, **p <0.01, ***p <0.001.

To further validate these observations, we measured IL-6 and TNF by ELISA in supernatants of THP-1 macrophages treated with Sm16 (34–117) alone, LPS alone and both together. Cells treated with Sm16 (34–117) alone did not secrete TNF but did secrete higher levels of IL-6 compared to untreated controls, although this increase was not statistically significant (Fig 6A). This weak pro-inflammatory effect of Sm16 (34–117) was also observed using BMDMs from C57/BL6 and Balb/c mice (S4 Fig). By contrast, LPS stimulation elicited a highly significant increase in levels of IL-6 and TNF secreted by THP-1 macrophages. Addition of Sm16 (34–117) to LPS-treated cells significantly reduced the amount of IL-6 and TNF released (Fig 6A).

Studies were performed with BMDMs from Balb/c mice to demonstrate that the effect of Sm16 was not restricted to THP-1 cells. In addition, to exclude the possibility of the anti-inflammatory effects of Sm16 (34–117) resulting from its direct binding to LPS (especially at high doses) we incubated BMDMs with Sm16 (34–117) at a range of concentrations (5–50 μg) for 30 min before washing the cells and subsequently adding LPS. Cell supernatants were collected following an overnight incubation and the quantities of TNF and IL6 in samples were measured by ELISA (Fig 6B). Sm16 (34–117) inhibited macrophage activation in a dose-dependent manner (5–50 μg/ml). Our data shows, therefore, that Sm16 can effectively modulate the inflammatory response of these murine macrophages and human THP-1 cells to stimulation with LPS.

To determine if the conserved α-helix region held the immune modulating activity of Sm16 and to identify a smaller effective anti-inflammatory peptide, we synthesised peptides corresponding to the following residues, Sm16 (52–77), Sm16 (60–80), Sm16 (73–107), Sm16 (85–96), Sm16 (85–115), or Sm16 (95–115), and tested these against THP-1 cells. We used IL-6 as our measure of blocking activity since microarray data showed that this cytokine was affected to a greater degree by Sm16 (52–77) than TNF (fold change of 309 vs 14.9, S3) and its secretion from LPS-stimulated THP-1 cells was effectively blocked by Sm16 (52–77) (Fig 6A). Compared to the parent Sm16 (34–117), none of these peptide derivatives significantly induced IL-6 secretion directly from THP-1 macrophages (Fig 6C). Moreover, no peptide significantly blocked the pro-inflammatory effect of LPS (Fig 6D).

### Changes to human macrophage gene expression exerted by Sm16 (34–117)

**Fig 7:**
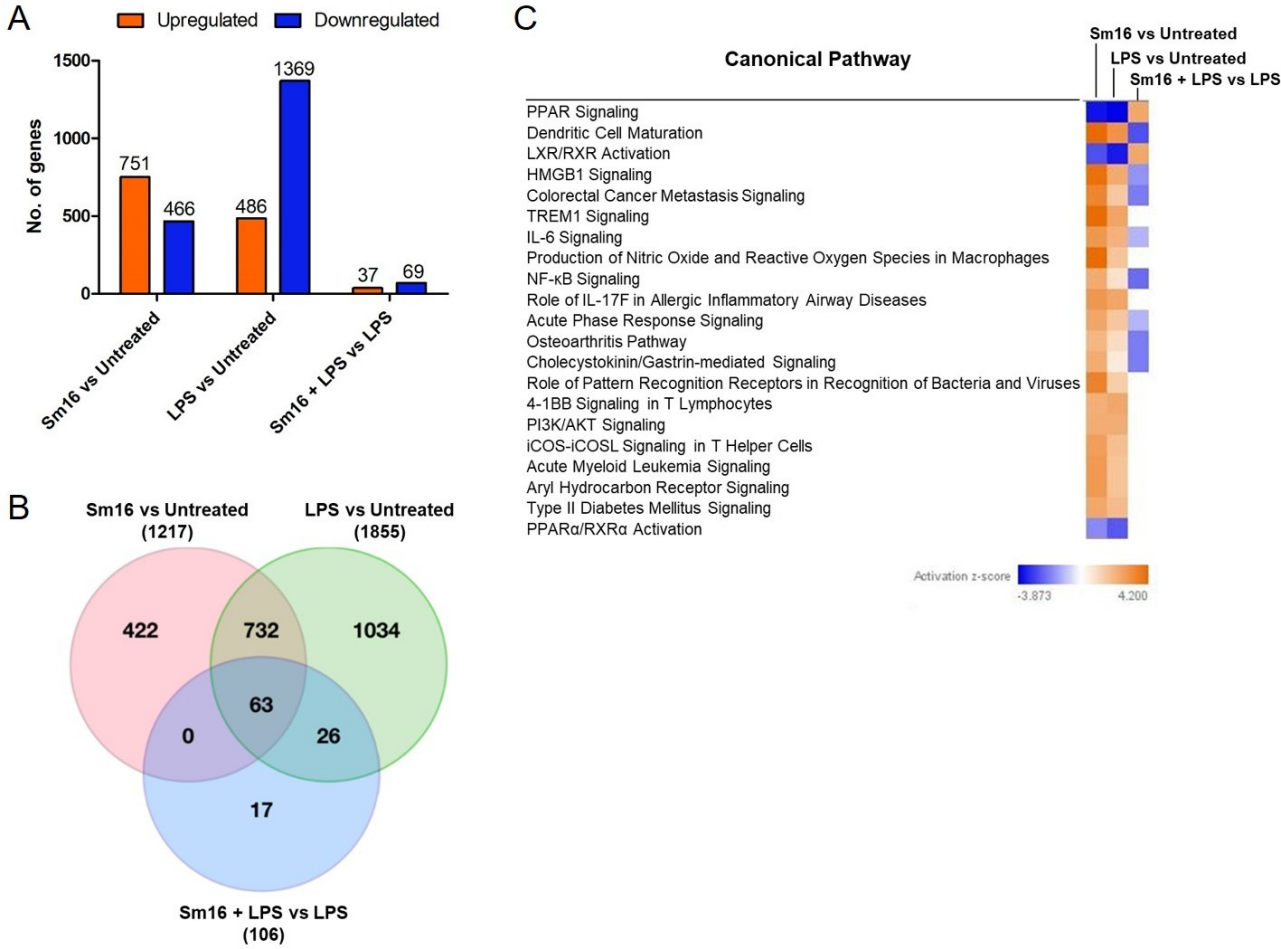
Sm16 (34–117) treatment significantly alters gene expression in THP-1 macrophages. THP-1 macrophages (2.5 x 10^5^) were treated with Sm16 (34–117) (20 μg/ml) and/or LPS (100 ng/ml) or not treated (Untreated) for 4 hrs before extracting RNA for analysis using Illumina HT12 V.4 Expression Bead Chips. Significantly (p <0.05) differentially expressed genes were identified by ANOVA when analysing Sm16 vs Untreated; LPS vs Untreated; Sm16 + LPS vs LPS alone. Data is derived from three independently performed experiments. (**A**) Overview of differential gene expression analyses detailing total number of genes that were up- and down-regulated >1.5 fold and <-1.5 fold, represented by orange and blue bars, respectively. (**B**) Venn diagram depicting overlap of differentially expressed genes across the respective analyses. (**C**) Canonical pathways predicted to be affected by the respective treatments as determined by IPA analysis of the differentially expressed genes (± 1.5 fold change). Inhibition and activation of pathways are shown by the z-score, represented by a scale of blue to orange, respectively.

To investigate the effects of Sm16 (34–117) on human macrophage gene transcription, THP-1 macrophages were incubated with Sm16 (34–117), LPS, or LPS and Sm16 (34–117) and mRNA transcripts analysed using Illumina HT12 V.4 Expression Bead Chips. In cells treated with Sm16 (34–117) only, transcription of a total of 1217 genes was significantly (p<0.05) changed: 751 gene transcripts exhibited increased expression (>1.5 fold) and 466 were down-regulated (<-1.5 fold) (Fig 7A; see S4 Table for top 70 genes differentially regulated by Sm16). LPS treatment significantly affected the transcription of 1855 genes, 486 of which showed increased expression while 1369 decreased (Fig 7A).

Of the 1217 genes for which expression was changed significantly by Sm16 (34–117), 65% (795) overlapped with the genes significantly changed by LPS stimulation. The directionality of the genes in this cohort was identical across the two sets of differential gene expression analyses, i.e. the same genes were up- or down-regulated in each group. Analysis of the remaining 35% (422) of genes that exclusively responded to Sm16 (34–117) revealed that these genes are most highly associated with cellular movement and development, inflammatory responses and tissue morphology. Based on their differential expression, IPA indicates that Sm16 is likely to cause an increase in lymphocyte populations, increase cell viability, cellular movement and phagocytosis, as well as a decrease in myeloid cell populations and inflammatory responses (S5 Fig).

THP-1 macrophages treated with LPS and Sm16 (34–117) showed transcriptional changes in only 106 genes compared to cells treated with LPS alone: of these, 37 genes showed >1.5 fold increased expression, while 69 <-1.5 fold decreased in expression (Fig 7A and 7C; see also S5 Table for top 70 genes differentially regulated by LPS followed by Sm16). A full list of the differential expression analyses results can be found in S6 Table.

Based on the differential changes to gene expression, Ingenuity Pathway Analysis (IPA) predicted that the pathways most negatively affected by treatment of the macrophages with either Sm16 (34–117) or LPS are nuclear receptors PPAR and LXR/RXR. These transcription factors are intricately involved in regulating cellular metabolism of macrophages (fatty acid, cholesterol and glucose homeostasis) and are central to the modulation of innate immune cell fate [38,39] (Fig 7C). However, when cells were first treated with LPS and then followed by Sm16 (34–117) both of these signaling pathways were up-regulated (Fig 7C). Conversely, several inflammatory signaling pathways including dendritic cell maturation, NF-κB signaling, HMGB1 signaling, acute phase responses, and IL-6 are putatively activated by Sm16 (34–117) and LPS alone, and are inhibited when cells are first treated with LPS and then with Sm16 (34–117) (Fig 7C).

The predicted implications of the changes to gene expression exerted by Sm16 (34–117) alone on the biological processes of macrophages include increased leukocyte activation and adhesion, chemotaxis, inflammatory responses and cell and organismal survival (S6 Fig). Sm16 (34–117), however, showed differences with LPS most obviously in its suppression of biological functions associated with morbidity/mortality and organismal death that were activated by LPS. These results further emphasise that while the Sm16 (34–117) itself can activate various inflammatory responses in macrophages it also has potent inhibitory activity against LPS-induced inflammation.

## Discussion

Phylogenetic, structural and functional analysis of the well-known schistosome-secreted molecule, Sm16, provides strong evidence for its inclusion within the helminth defence molecule (HDM) family of immunomodulators. Previously, our clustal analysis of several members of HDMs suggested an evolutionary link between Sm16 and HDMs [40]. Given the extensive range of genomic data now available for helminth species, a more thorough phylogenetic analysis was carried out and confirmed these previous findings. Gene structure analysis further supported the expansion of this family of Sm16-like molecules by demonstrating a conserved intron-exon pattern amongst the HDM and Sm16 genes.

Furthermore, we found that Sm16-like HDMs form a distinct branch of the HDMs specific to the Schistosomatoidea superfamily which is consistent with the early evolutionary divergence of this superfamily from the other trematode families [41]. Sequence alignments of Sm16 homologues in *S*. *japonicum*, *S*. *haematobium*, *S*. *curassoni*, *S*. *margrebowiei*, *S*. *mattheei*, *S*. *rodhaini*, and *Trichobilharzia regenti*, showed that Sm16-like molecules are structurally highly conserved within this superfamily. Since the Schistosomatoidea superfamily also express members of the Fasciola-like HDMs it is clear that the two branches arose from a common ancestral HDM. Therefore, these analyses verify the view that the Sm16-like HDMs diverged to perform a function(s) that is unique to Schistosomatoidea, most obviously, a role in the process of skin invasion by cercariae which is unique to this trematode superfamily.

Looking more broadly, our genomic searches also discovered that the HDM family of molecules are exclusively present in the genomes of trematode species. All trematode genomes examined possessed at least one HDM-encoding gene whereas these were absent from all nematode and cestode genomes. Such conservation within trematode species indicates that HDM molecules are of great importance to the development and/or survival of these digenean endoparasites. Our studies with *F*. *hepatica* (FhHDM/FhMF6p) have suggested that trematodes secrete HDMs to modulate the host immune responses to ensure their longevity, possibly by preventing the activation of pro-inflammatory responses via the inflammasome [42]. Another idea proposed by Martinez-Sernandez et al. [43] relates to the heme-binding property of FhHDM/FhMF6p and suggests that they play a role in the scavenging of potentially damaging free heme released from haemoglobin during feeding by the parasites.

Structural analysis of the Sm16 protein demonstrates that it is primarily an α-helical molecule. We highlighted the presence of four consecutive hydrophobic faces in the major α-helical region that spans much of the Sm16 C-terminal section (residues 52–115). Hydrophobic residues were concentrated on one face of each α-helix and indicate that Sm16 is considerably amphipathic. This shows that the structural and biochemical properties of the Sm16-like and Fasciola-like HDMs are also very similar in that they are α-helical, amphipathic, and cationic [24]. Indeed, the integrity of the C-terminal sequence and structure of these molecules appears to be inherently important for their immunomodulatory activity. Truncation or disruption of the Sm16 sequence at the C-terminus impairs its ability to bind to surface membranes and to be internalised by mammalian cells [14,20,44], which has also been observed in our studies on *F*. *hepatica* FhHDM/FhMF6p [36].

The first studies of Sm16 two decades ago found that it was expressed exclusively by sporocysts, cercariae, and early schistosomulae of *S*. *mansoni* [9,10]. We also found that Sm16 constitutes a considerable proportion of the proteins in cercariae and, in keeping with the proteomic studies by Curwen et al. [45], is secreted during the mechanical transformation of cercariae to schistosomulae. The molecule is stored in abundance within the acetabular glands and rapidly expelled from these during skin penetration [45,46]; however, this transient expression and secretion into the host tissues is clearly insufficient to induce detectable antibodies in the early weeks following a primary infection. Most reports agree that Sm16 is not expressed by adult worms [9–12,46]. Although we detected low levels of Sm16 expression in female adult and mixed-adult extracts by qPCR in this study, we presume that this is due to some residual presence of eggs in adult female worms as no expression was found in male worms.

Our finding that Sm16 is expressed in eggs disagrees with most earlier studies and the more recent report by Bernardes et al. [12]. The discrepancy may be because we employed more sensitive methods of Western blots (chemiluminescence) and gene amplification by qPCR. This would also explain why our results are consistent with studies by Gobert et al. [11] who showed using microarrays that *S*. *japonicum* Sj16 is enriched in eggs. Our immunohistochemistry studies also clearly showed the presence of Sm16 within the unembryonated miracidium. This raises the possibility that Sm16 is involved in egg-driven immunomodulation and while we did not observe Sm16 in the tissues surrounding the egg, the presence of a signal peptide in the molecule and antibodies to Sm16 in the blood of infected mice suggests that it is secreted. Although antibodies were not detected until sometime after week 10 post-infection this could be because the molecule is secreted in low levels, is poorly immunogenic due to its small size, or is secreted late in the entrapped egg. Also, there may be little or no response to Sm16 until the host immune system is exposed to the increasing number of eggs released by females or when tissue-lodged eggs die and degrade and their contents disperse into the tissues. Nevertheless, our studies encourage future investigations to determine if Sm16 plays a role in egg-induced inflammation, in down-modulating the egg granuloma (which occurs between 8–20 weeks after infection) and/or in facilitating the immune-dependent exit of eggs through the intestine [47].

Much of the research to date that has evaluated the function of Sm16 has been conducted using a recombinant formulation expressed in *E*. *coli* that either features a mutation with two alanine substitutions at positions 92 and 93 [13,14,44] or a truncation of the last 27 C-terminal residues [12]. These modifications were made due to the inability to express soluble recombinant Sm16 in this prokaryotic system, perhaps owing to the amphipathic nature of the C-terminal section of the protein. However, we would argue that these modifications also compromised the native structure of the molecule and, more importantly, its immunological function since these alterations were made within the region that is critical for the immunomodulatory activity of HDM. Indeed, Robinson et al. [36] demonstrated that disruption of the C-terminal amphipathic α-helical by substitution of a leucine for a proline resulted in its inability to bind lipid membranes and inhibit vacuolar ATPase. We report here that full-length Sm16 can be expressed and secreted in the eukaryotic methylotrophic yeast *P*. *pastoris* and that this recombinant, as well as a synthetic version, bound to macrophages and was endocytosed into the endosomal/lysosomal system like other HDMs [36]. Bernardes et al. [12] acknowledged that the failure of their recombinant Sm16 vaccine to promote parasite elimination could have been because it lacked the C-terminal 27 residues; therefore, a repeat of these trials with yeast-expressed or synthetic full-length Sm16 may be worthwhile.

Another anomaly we found between our studies and previously reported work regards the sequence identities between the Sm16 of *S*. *mansoni* and the homologs found in *S*. *japoncium*. In the report of the discovery of the Sj16 homolog in *S*. *japonicum*, Hu et al. [18] states that this molecule ‘shares 99% identity with Sm16 in its nucleotide sequence, and 100% identity in its protein sequence’. A recombinant formulation of the molecule was produced, termed rSj16, and has been used in a number of studies [18–23, 48–52]. We show here with our in-depth analysis of the genomic data currently available that while Sm16 represents a single copy gene in *S*. *mansoni*, three Sm16-like molecules exist in the genome of *S*. *japonicum*; however, none of the three Sj16s share 100% primary sequence identity with Sm16. The percentage identities of Sj16_1, Sj16_2 and Sj16_3 compared to Sm16 are 66%, 63% and 38%, respectively.

We opted to evaluate the bioactive properties of a chemically synthesized Sm16 as we have previously shown that HDMs bind LPS very strongly in solutions making it difficult to isolate them free of endotoxin [24]. The LPS-binding capacity of HDM have also been reported by Martinez-Sernandez et al. [43,53] and Kang et al. [54]. Chemically synthesized peptides have various production benefits compared to recombinantly-produced peptides including reduced costs, capacity to up-scale, increased purity, and are endotoxin free. Here we show that Sm16 (34–117) is readily internalized by the endocytic/lysosomal system of macrophages and causes significant changes to the transcription of genes that are primarily associated with immune responses. Macrophages are key players in the innate immune response to pathogens and are also pivotal in coordinating tissue repair [55,56]. In the early stages of infection innate immune responses are potently stimulated by schistosomes and typically a Th1-type inflammatory response is mounted by the host [6]. In light of our observations that Sm16 exhibits weak pro-inflammatory activity, these responses could be associated in part with the early and rapid release of an abundance of this molecule. Upon infection, schistosome larvae induce IL-12p40 secretion from dendritic cells and macrophages, a cytokine considered to be a key mediator of the cutaneous inflammation [57]. Furthermore, radiation-attenuated cercariae, which have a delayed migration through the skin, elicit an IL-12p40-mediated Th1 response that confers protection against further parasite invasion [58]. The treatment of macrophages with Sm16 (34–117) resulted in a 1.5-fold increase (p = 0.03) in IL-12p40 transcripts (IL12B; S6 Table) which would support the idea that Sm16 secreted by schistosomulae during infection could contribute to the IL-12p40-mediated inflammatory response. In addition, it has been suggested that IL-12p40 also has the propensity to inhibit eosinophilia [59], which may facilitate unimpeded access for the worm into host vasculature. This weak but significant pro-inflammatory property of Sm16-like HDMs has been previously overlooked in studies of its immunomodulatory activity as experiments involving macrophages treated with Sm16 alone were not performed or reported [14,18].

Sm16 (34–117) attenuated the pro-inflammatory responses of LPS-stimulated macrophages compared to LPS controls in a dose-dependent manner. This observation suggested that Sm16 (34–117) exposure arrests macrophage responses to TLR4 activation and is supported by the anti-inflammatory activity of Sm16-derived molecules, and other HDMs, in dampening responses to LPS [13,14,17,19,20,22,24,26,40,50–52]. Our results also confirm that the chemically synthesized Sm16 (34–117) retains the anti-inflammatory properties of Sm16 (and also binds anti-Sm16 antibodies in infected mice blood). However, an attempt to identify a shorter peptide sequence with similar activity to the parent molecule activity, albeit focused around the α-helical hotspots in the C-terminal region, was not successful and suggests that the intrinsic property of the Sm16 to be taken up by cells and alter their transcriptional profile is dependent on several conjoined motifs. However, in light of the immunotherapeutic potential of Sm16, we have established that the synthetic Sm16 (34–117) is bioactive and can be used in future studies to elucidate Sm16 function as well as being a cost-effective option for further bio-therapeutics development.

Analysis of cytokine production by human acute monocytic leukaemia THP-1 macrophages stimulated with Sm16 and with LPS showed that both induced pro-inflammatory responses, although the latter exhibit far higher potency. Microarray analysis of these cells found that of the 1217 genes that showed a significant change in expression when stimulated with Sm16 (34–117), 65% (795) overlapped with the genes also significantly changed by LPS stimulation, with comparable up or down expression of genes. However, Sm16 exclusively altered the expression of 422 genes (35%) that were most highly associated with cellular movement and development, inflammatory responses and tissue morphology, and according to Ingenuity Pathway Analysis (IPA) were likely to elicit an increase in lymphocyte populations, increase cell viability, cellular movement and phagocytosis, in addition to decreasing myeloid cell populations and inflammatory responses. The data therefore indicates that while Sm16 (34–117) displays pro-inflammatory activity with similarities to LPS its effect on macrophage cell activation and signalling was distinct.

Interrogation of the RNA microarray data of Sm16 (34–117)-treated THP-1 macrophages suggested that at least one mechanism utilised by Sm16 to regulate the response of macrophages to activation by inflammatory ligands (such as LPS) was via the control of ligand-activated transcription factors PPAR and LXR. These nuclear receptors compete to hetero-dimerise with RXR before binding to DNA response elements in the promoter regions of target genes that control macrophage lipid, cholesterol and glucose homeostasis [38,39]. PPAR/LXR are expressed by a wide range of hematopoietic immune cells, including macrophages, and are known to have immunosuppressive effects on both the innate and adaptive arms of the mammalian immune response. They can alter gene expression to inhibit inflammatory cytokine transcription and the development of CD4+ T cells, and have also been linked to parasite-mediated immune modulation [60]. In this study, PPAR/LXR signaling was activated when the human macrophages were treated with the combination of LPS and Sm16 (34–117) which could suggest that this is a mechanism through which the peptide exerts its anti-inflammatory effects. Interestingly, using microarrays, Tanaka et al. [26] recently showed that blocking of LPS-induced inflammatory responses in murine (Balb/c) bone-marrow derived macrophages by a synthetic *F*. *hepatica* HDM also involved in the activation of PPAR/LXR signaling. *In vivo* experiments performed by Wang et al. [23] showed that rSj16 delivery protected mice from DSS-induced colitis which correlated with the inhibition of PPAR-α signaling in the colon. Therefore, further investigation into the intricacies of Sm16 control of PPAR/LXR signaling, the implications of its effects on inflammatory responses, and indeed the affected cell-types that orchestrate the immunomodulation *in vivo* is warranted.

The secretion of antigens by helminth parasites may inhibit endotoxin-induced inflammation to dampen Th1-type responses and indirectly promote a Th2 environment in which endoparasitic helminths can thrive [61–63]. However, the immune system modulation/polarisation exerted by flatworms and other helminth infections can leave hosts more susceptible to secondary infections that could potentially be deleterious for both the host and parasite [64–66]. We have suggested that dampening classical immune activation by endotoxin with secreted molecules could be a mechanism employed by trematodes like *F*. *hepatica* and *S*. *mansoni* to confer tolerance to secondary bacterial infections [67]. A feature of these infections is the disruption of anatomical barriers, either at the skin, intestine, bladder or bile ducts which could lead to the translocation of bacteria into the host circulation and cause septicaemia and septic shock. Indeed, a study has shown that systemic endotoxin levels in individuals with schistosomiasis were extremely high, notably higher than lethal endotoxin levels reported in cases of septic shock [68]. Accordingly, secretion of HDM by these flatworms may be important in sustaining a general dampening of pro-inflammatory responses to co-infection with microbial pathogens, possibly via activation of PPAR and LXR/RXR transcription factors.

In conclusion, we have shown that Sm16 and its homologues within the Schistosomatoidea superfamily are distinct members of the HDM family of short secretory peptides that are expressed exclusively by trematode species. Thus, our studies elevate the general importance of HDMs as a *bone fide* family of immunomodulatory molecules in these globally important parasites of humans and their livestock. In the context of the collective published data, our study broadens our understanding of Sm16-like molecules and supports the idea that they play an important role in key host-parasite interactions including the scavenging/detoxification of haemoglobin-derived heme and iron transport [43,53] while also advancing the proposal that the secretion of Sm16 by eggs could contribute to disease pathogenesis and/or transmission. However, further research, for example through specific gene knock-down and/or gene editing, would go a long way towards elucidating the true importance of Sm16 in schistosomiasis. Finally, as we have shown that a synthetic form of this molecule, Sm16 (34–117), retains bioactive and immunomodulatory properties which augers well for the future pursuit of cost-effective trematode-derived immune-therapeutics.

## Supporting information

S1 Fig Structural analyses of the Schistosomatidae-specific family of Sm16-like molecules. (**A**) A MAFFT amino acid alignment of the Sm16-like proteins from trematodes. The predicted signal peptide is shown underlined and in italics. The black line depicts the area of the Sm16-like molecules that is amphipathic. The four colour blocks represent the sequence encoded by the four exons depicted in the genomic organisation below. (**B**) Schematic representation of the genomic organisation of the Sm16-like molecules. Exons and introns are represented as coloured boxes and lines, respectively. The numbers denote the number of nucleotide base pairs. ^Sr16 gene–Part of the last exon is missing due to an error in the *Schistosoma rodhaini* genome scaffold. *Sh16 gene–The second intron cannot be determined within the current *Schistosoma haematobium* genome assembly; currently the first two exons are present on the forward DNA strand, with the remaining part of the gene present on the opposite strand of the scaffold. ±As the Sj16_2 and Tr16_2 genes are present at the beginning of their respective scaffolds the first exon cannot be determined within the current genome assemblies.
(TIF)

S2 Fig Structural analyses of the Trematode-specific family of Fasciola-like HDM molecules. (**A**) A MAFFT amino acid alignment of the Fasciola-like HDM proteins. The predicted signal peptide is shown underlined and in italics. The four colour blocks represent the sequence encoded by the four exons depicted in the genomic organisation below. (**B**) Schematic representation of the genomic organisation of the Fasciola-like HDM molecules. Exons and introns are represented as coloured boxes and lines, respectively. The numbers denote the number of nucleotide base pairs. ^As the TrHDM gene is present at the beginning of the genomic scaffold the first exon cannot be determined within the current genome assemblies.
(TIF)

S3 Fig Purification of yeast-expressed recombinant Sm16. Top: gene accession numbers of Sm16/SPO-1 and primary sequence. The signal sequence is shaded in black. The DNA sequence encoding Sm16 without the signal sequence was cloned into a pPinkα-HC vector and expressed in *Pichia pastoris* as a secreted 6xHis-tagged protein. Recombinant Sm16 was purified using Ni^2+^-affinity chromatography and analysed on a 16% SDS-PAGE electrophoresis gel which was subsequently stained with Coomassie blue. Sm16 was also detected using anti-His tag and anti-Sm16 antibodies.
(TIF)

S4 Fig Pro-inflammatory effect exerted by Sm16 (34–117) on murine bone-marrow derived macrophages (BMDMs). BMDMs from (A-B) C57/BL6 and (C-D) Balb/c mice were treated with 20 μg/ml of Sm16 or untreated (Unstim) for 24 hrs. (A, C) KC, and (B, D) IL-6 levels in cell supernatants were measured by ELISA. Data are presented as the mean and SEM of three independent experiments analysed using unpaired t-tests. Significance indicated compared to unstimulated controls. (*p <0.05, ***p <0.001).
(TIF)

S5 Fig Biological processes associated with genes independently affected by Sm16. IPA of 422 genes differentially up- regulated >1.5 fold (p <0.05) in macrophages by treatment with Sm16 and independent of genes associated with the cellular response to LPS, represented as log p value. The orange line highlights the threshold of–log(0.05) / 1.3.
(TIF)

S6 Fig Comparative analyses of the biological effects exerted by Sm16 (34–117) and LPS as shown by differential gene expression. THP-1 macrophages (2.5 x 10^5^) were untreated or treated with Sm16 (34–117) alone (20 μg/ml), LPS alone (100 ng/ml) or with both Sm16 (34–117) and LPS for 4 hrs before extracting RNA for analysis using Illumina HT12 V.4 Expression Bead Chips. Significantly differentially expressed genes were identified by ANOVA and IPA analysis of these produced predicted effects on associated functions. Inhibition and activation of pathways are shown by the z-score, represented by a scale of blue to orange, respectively.
(TIF)

S1 Table Accession number/protein identifiers of the sequences used for the phylogenetic analysis. (DOCX)

S2 Table Details of parasite genome databases and seed sequences used for BLAST analysis. (DOCX)

S3 Table Cytokine array analysis of supernatants of THP-1 macrophages that were untreated or treated with Sm16 (34–117), LPS or LPS and Sm16 (34–117). Numbers represent fold change in cytokine signal. Signal intensity was measured by densitometry. When comparing separate membranes values were normalised using a comparative ratio calculated using densitometry values for membrane positive control spots.
(DOCX)

S4 Table Top 70 genes differentially regulated by adding Sm16 to THP-1 macrophages. (DOCX)

S5 Table Top 70 genes differentially regulated by adding Sm16 to LPS-treated THP-1 macrophages. (DOCX)

S6 Table Differential expression analyses by microarray of THP-1 macrophages treated with Sm16 and LPS. (XLSX)
