# Supplementary figures and images for: Schistosoma mansoni immunomodulatory molecule Sm16/SPO-1/SmSLP is a member of the trematode-specific helminth defence molecules (HDMs)
Source: PLoS Negl Trop Dis. 2020 Jul 9;14(7):e0008470. doi: 10.1371/journal.pntd.0008470 (PMC7373315; doi:10.1371/journal.pntd.0008470)

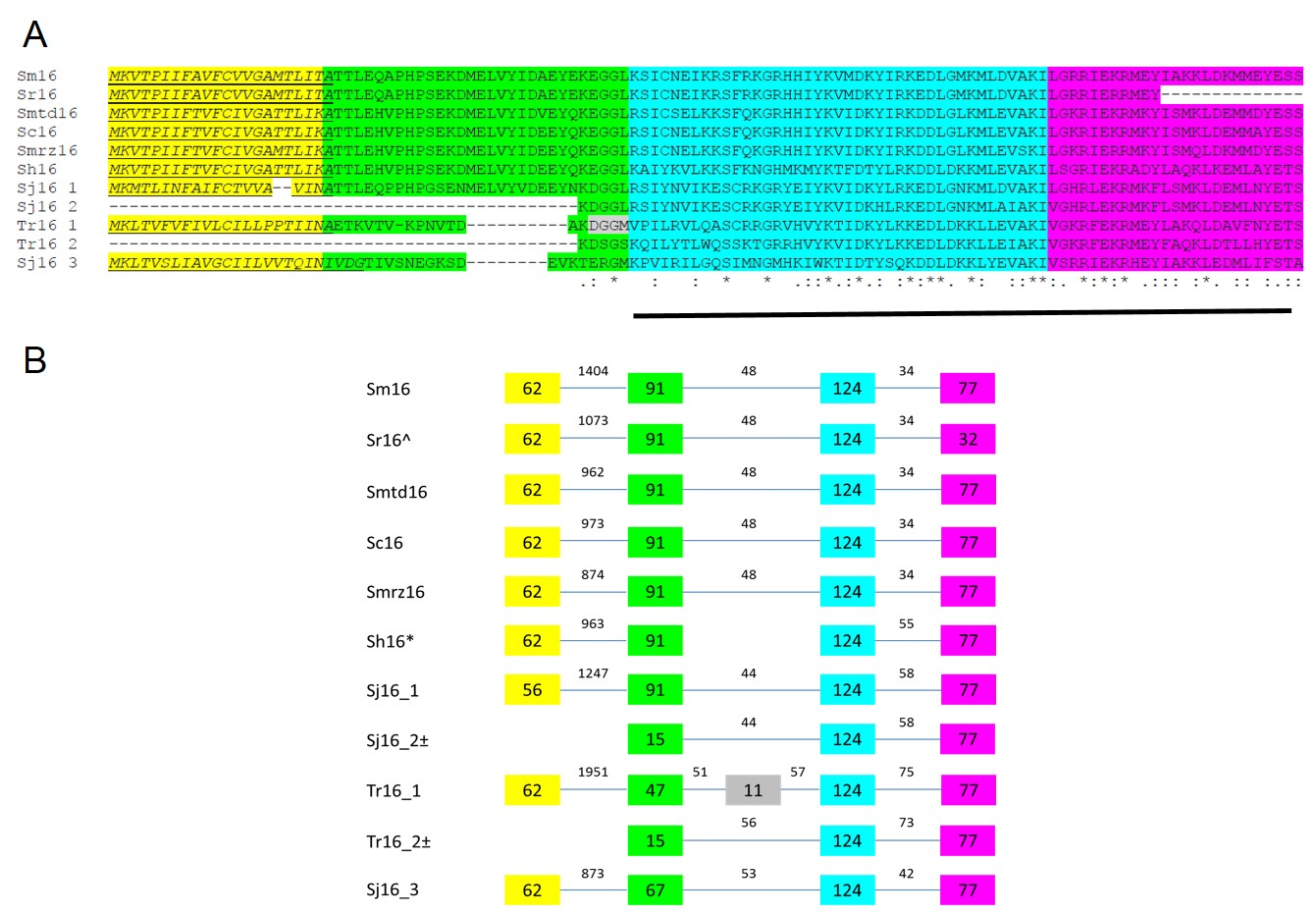

Supplement: S1 Fig — (A) A MAFFT amino acid alignment of the Sm16-like proteins from trematodes. The predicted signal peptide is shown underlined and in italics. The black line depicts the area of the Sm16-like molecules that is amphipathic. The four colour blocks represent the sequence encoded by the four exons depicted in the genomic organisation below. (B) Schematic representation of the genomic organisation of the Sm16-like molecules. Exons and introns are represented as coloured boxes and lines, respectively. The numbers denote the number of nucleotide base pairs. ^Sr16 gene–Part of the last exon is missing due to an error in the Schistosoma rodhaini genome scaffold. *Sh16 gene–The second intron cannot be determined within the current Schistosoma haematobium genome assembly; currently the first two exons are present on the forward DNA strand, with the remaining part of the gene present on the opposite strand of the scaffold. ±As the Sj16_2 and Tr16_2 genes are present at the beginning of their respective scaffolds the first exon cannot be determined within the current genome assemblies. (TIF) [file pntd.0008470.s001.tif]

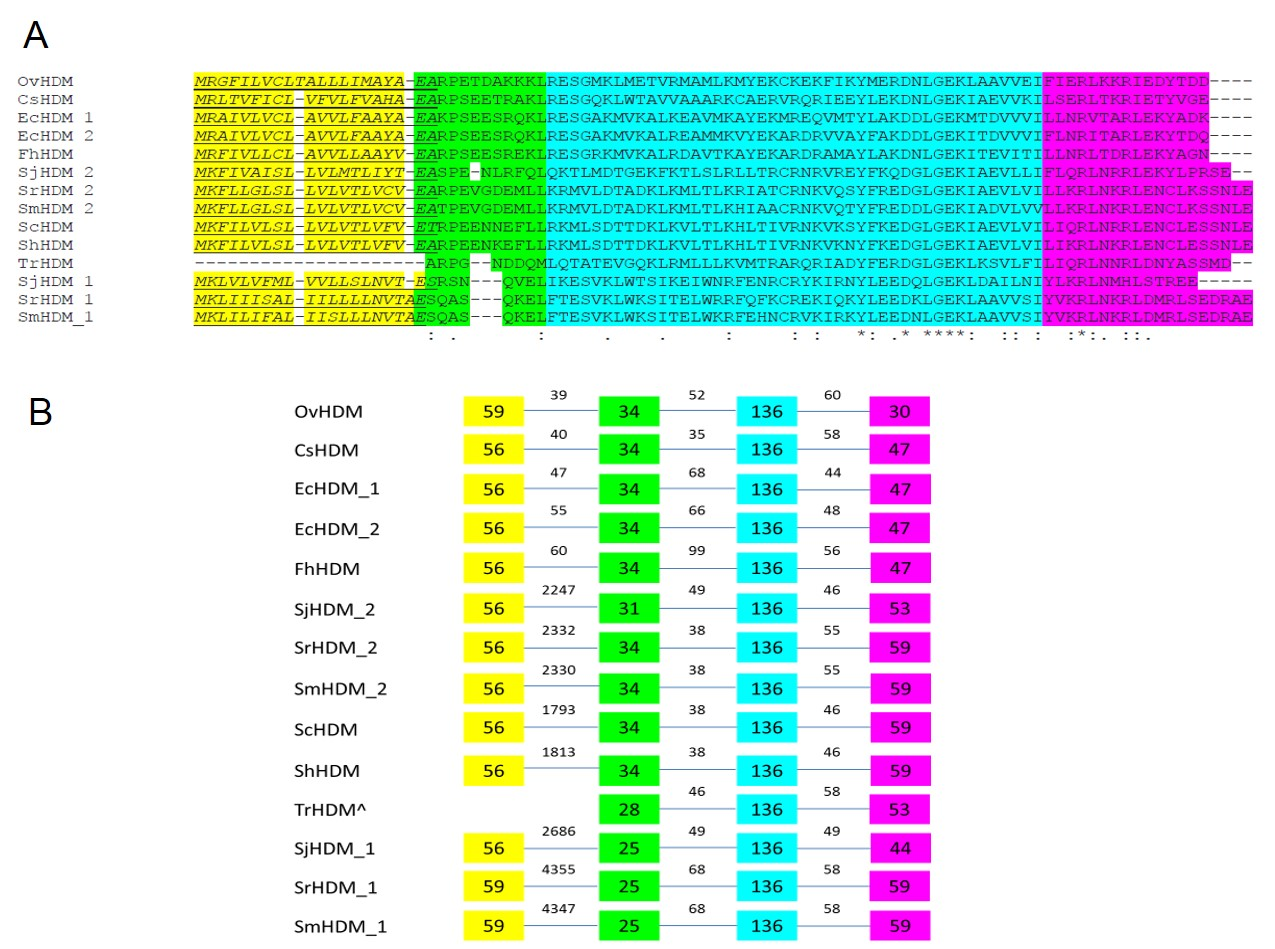

Supplement: S2 Fig — (A) A MAFFT amino acid alignment of the Fasciola-like HDM proteins. The predicted signal peptide is shown underlined and in italics. The four colour blocks represent the sequence encoded by the four exons depicted in the genomic organisation below. (B) Schematic representation of the genomic organisation of the Fasciola-like HDM molecules. Exons and introns are represented as coloured boxes and lines, respectively. The numbers denote the number of nucleotide base pairs. ^As the TrHDM gene is present at the beginning of the genomic scaffold the first exon cannot be determined within the current genome assemblies. (TIF) [file pntd.0008470.s002.tif]

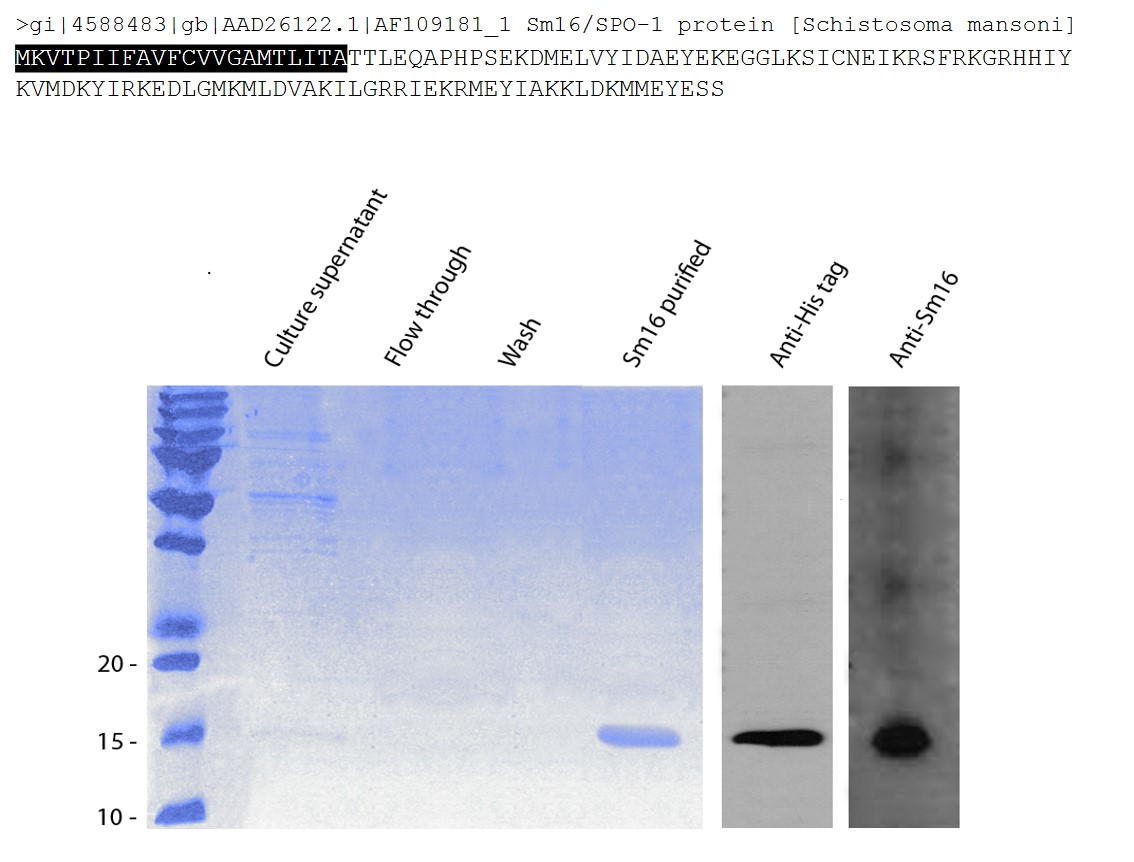

Supplement: S3 Fig — Top: gene accession numbers of Sm16/SPO-1 and primary sequence. The signal sequence is shaded in black. The DNA sequence encoding Sm16 without the signal sequence was cloned into a pPinkα-HC vector and expressed in Pichia pastoris as a secreted 6xHis-tagged protein. Recombinant Sm16 was purified using Ni2+-affinity chromatography and analysed on a 16% SDS-PAGE electrophoresis gel which was subsequently stained with Coomassie blue. Sm16 was also detected using anti-His tag and anti-Sm16 antibodies. (TIF) [file pntd.0008470.s003.tif]

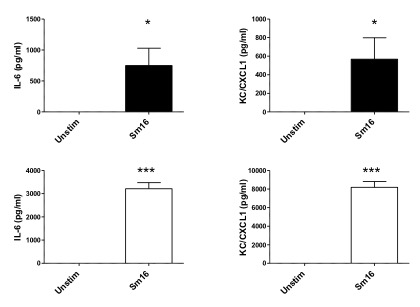

Supplement: S4 Fig — BMDMs from (A-B) C57/BL6 and (C-D) Balb/c mice were treated with 20 μg/ml of Sm16 or untreated (Unstim) for 24 hrs. (A, C) KC, and (B, D) IL-6 levels in cell supernatants were measured by ELISA. Data are presented as the mean and SEM of three independent experiments analysed using unpaired t-tests. Significance indicated compared to unstimulated controls. (*p <0.05, ***p <0.001). (TIF) [file pntd.0008470.s004.tif]

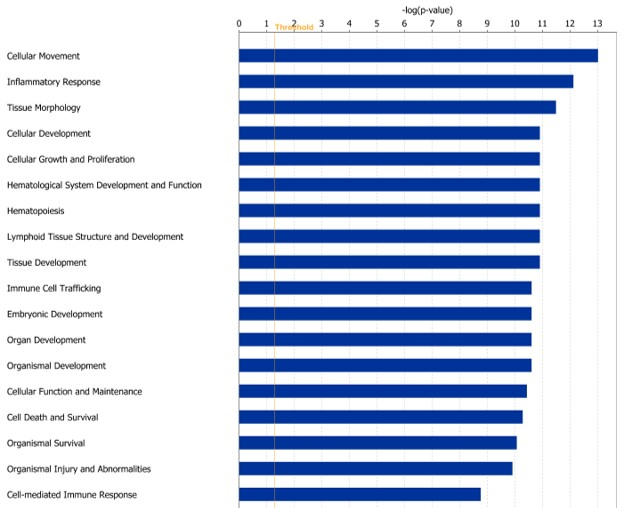

Supplement: S5 Fig — IPA of 422 genes differentially up- regulated >1.5 fold (p <0.05) in macrophages by treatment with Sm16 and independent of genes associated with the cellular response to LPS, represented as log p value. The orange line highlights the threshold of–log(0.05) / 1.3. (TIF) [file pntd.0008470.s005.tif]

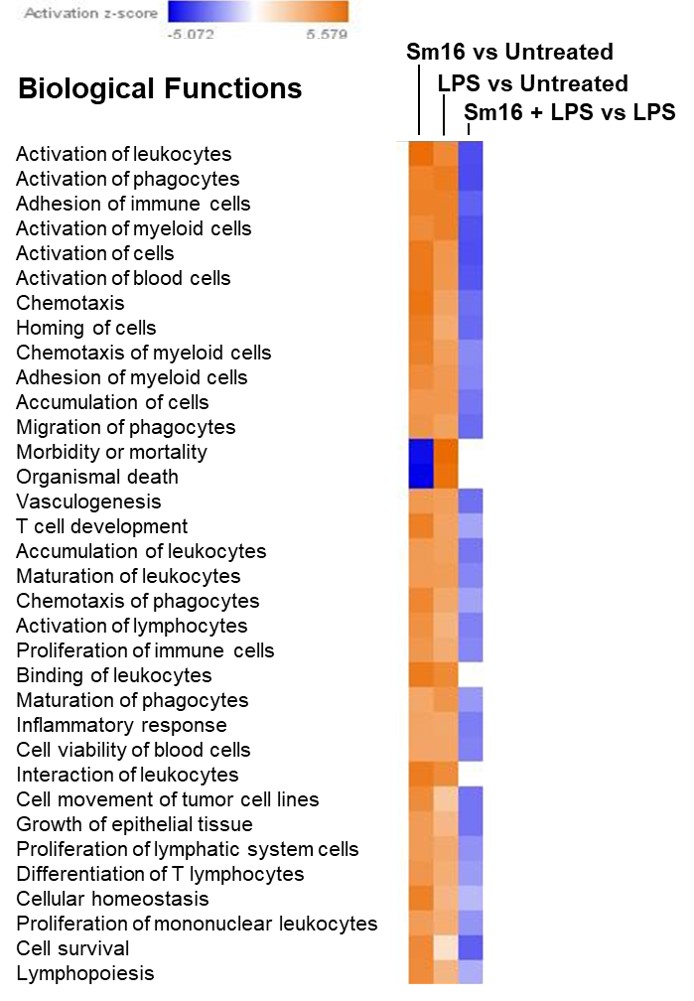

Supplement: S6 Fig — THP-1 macrophages (2.5 x 10^5^) were untreated or treated with Sm16 (34–117) alone (20 μg/ml), LPS alone (100 ng/ml) or with both Sm16 (34–117) and LPS for 4 hrs before extracting RNA for analysis using Illumina HT12 V.4 Expression Bead Chips. Significantly differentially expressed genes were identified by ANOVA and IPA analysis of these produced predicted effects on associated functions. Inhibition and activation of pathways are shown by the z-score, represented by a scale of blue to orange, respectively. (TIF) [file pntd.0008470.s006.tif]
