# Supplementary material for: Schistosoma mansoni immunomodulatory molecule Sm16/SPO-1/SmSLP is a member of the trematode-specific helminth defence molecules (HDMs)
Source: PLoS Negl Trop Dis. 2020 Jul 9;14(7):e0008470. doi: 10.1371/journal.pntd.0008470 (PMC7373315; doi:10.1371/journal.pntd.0008470)
Supplement: S1 Table — (DOCX) [file pntd.0008470.s007.docx]

**S1 Table: Accession number/protein identifiers of the sequences used for the phylogenetic analysis**

| **Nomenclature on Phylogram** | **Species** | **Accession/Protein Identifier** |
| --- | --- | --- |
| **Fasciola-like HDM** |  |  |
| CsHDM | *Clonorchis sinensis* | csin111951 / AAM55183 |
| EcHDM_1 | *Echinostoma caproni* | ECPE_0000705101 |
| EcHDM_2 | *Echinostoma caproni* | ECPE_0000435301 |
| FhHDM | *Fasciola hepatica* | BN1106_s2101B000084 / CCA61804 |
| OvHDM | *Opisthorchis viverrini* | T265_03708 |
| ScHDM | *Schistosoma curassoni* | SCUD_0000854001 |
| ShHDM | *Schistosoma haematobium* | MS3_06298 |
| SjHDM_1 | *Schistosoma japonicum* | Sjp_0016090 |
| SjHDM_2 | *Schistosoma japonicum* | Sjp_0006990 |
| SmHDM_1 | *Schistosoma mansoni* | Smp_194860 |
| SmHDM_2 | *Schistosoma mansoni* | Smp_096790 |
| SrHDM_1 | *Schistosoma rodhaini* | SROB_0000777001 |
| SrHDM_2 | *Schistosoma rodhaini* | SROB_0001573801 |
| TrHDM | *Trichobilharzia regenti* | TRE_0000729501 |
| **Sm16-like HDM** |  |  |
| Sc16 | *Schistosoma curassoni* | SCUD_0001195101 |
| Sh16 | *Schistosoma haematobium* | MS3_06289 / MS3_06291 |
| Sj16_1 | *Schistosoma japonicum* | Sjp_0006960 |
| Sj16_2 | *Schistosoma japonicum* | Sjp_0006970 |
| Sj16_3 | *Schistosoma japonicum* | Sjp_0006980 |
| Sm16 | *Schistosoma mansoni* | Smp_341790 / AAD26122 |
| Smrz16 | *Schistosoma margrebowiei* | SMRZ_0000959601 |
| Smtd16 | *Schistosoma mattheei* | SMTD_0000820701 |
| Sr16 | *Schistosoma rodhaini* | SROB_0001216901 |
| Tr16_1 | *Trichobilharzia regenti* | TRE_0000474601 |
| Tr16_2 | *Trichobilharzia regenti* | TRE_0001421101 |
