# Supplementary material for: Schistosoma mansoni immunomodulatory molecule Sm16/SPO-1/SmSLP is a member of the trematode-specific helminth defence molecules (HDMs)
Source: PLoS Negl Trop Dis. 2020 Jul 9;14(7):e0008470. doi: 10.1371/journal.pntd.0008470 (PMC7373315; doi:10.1371/journal.pntd.0008470)
Supplement: S2 Table — (DOCX) [file pntd.0008470.s008.docx]

**S2 Table: Details of parasite genome databases and seed sequences used for BLAST analysis**

| **Parasite genome databases (WormBase ParaSite; WBPS11)** | | | |
| --- | --- | --- | --- |
| **Phylum Nematoda** | | **Phylum Platyhelminthes** | |
| **Species Name** | **BioProject ID** | **Species Name** | **BioProject ID** |
| *Acanthocheilonema viteae* | PRJEB4306 | *Clonorchis sinensis* | PRJDA72781 |
| *Ancylostoma duodenale* | PRJNA72581 | *Dibothriocephalus latus* | PRJEB1206 |
| *Angiostrongylus cantonensis* | PRJEB493 | *Echinococcus canadensis* | PRJEB8992 |
| *Anisakis simplex* | PRJEB496 | *Echinococcus granulosus* | PRJEB121 |
| *Ascaris lumbricoides* | PRJEB4950 | *Echinococcus granulosus* | PRJNA182977 |
| *Brugia malayi* | PRJNA10729 | *Echinococcus multilocularis* | PRJEB122 |
| *Bursaphelenchus xylophilus* | PRJEA64437 | *Echinostoma caproni* | PRJEB1207 |
| *Caenorhabditis elegans* | PRJNA13758 | *Fasciola hepatica* | PRJEB6687 |
| *Cylicostephanus goldi* | PRJEB498 | *Hydatigera taeniaeformis* | PRJEB534 |
| *Dictyocaulus viviparus* | PRJEB5116 | *Hymenolepis diminuta* | PRJEB507 |
| *Dictyocaulus viviparus* | PRJNA72587 | *Hymenolepis microstoma* | PRJEB124 |
| *Dirofilaria immitis* | PRJEB1797 | *Hymenolepis nana* | PRJEB508 |
| *Dracunculus medinensis* | PRJEB500 | *Macrostomum lignano* | PRJNA284736 |
| *Elaeophora elaphi* | PRJEB502 | *Macrostomum lignano* | PRJNA371498 |
| *Enterobius vermicularis* | PRJEB503 | *Mesocestoides corti* | PRJEB510 |
| *Globodera pallida* | PRJEB123 | *Opisthorchis viverrini* | PRJNA222628 |
| *Gongylonema pulchrum* | PRJEB505 | *Protopolystoma xenopodis* | PRJEB1201 |
| *Haemonchus contortus* | PRJEB506 | *Schistocephalus solidus* | PRJEB527 |
| *Haemonchus contortus* | PRJNA205202 | *Schistosoma curassoni* | PRJEB519 |
| *Heligmosomoides polygyrus* | PRJEB1203 | *Schistosoma haematobium* | PRJNA78265 |
| *Heligmosomoides polygyrus* | PRJEB15396 | *Schistosoma japonicum* | PRJEA34885 |
| *Heterorhabditis bacteriophora* | PRJNA13977 | *Schistosoma mansoni* | PRJEA36577 |
| *Litomosoides sigmodontis* | PRJEB3075 | *Schistosoma margrebowiei* | PRJEB522 |
| *Loa loa* | PRJNA246086 | *Schistosoma mattheei* | PRJEB523 |
| *Loa loa* | PRJNA60051 | *Schistosoma rodhaini* | PRJEB526 |
| *Meloidogyne incognita* | PRJEB8714 | *Schmidtea mediterranea* | PRJNA12585 |
| *Necator americanus* | PRJNA72135 | *Spirometra erinaceieuropaei* | PRJEB1202 |
| *Nippostrongylus brasiliensis* | PRJEB511 | *Taenia asiatica* | PRJEB532 |
| *Oesophagostomum dentatum* | PRJNA72579 | *Taenia asiatica* | PRJNA299871 |
| *Onchocerca volvulus* | PRJEB513 | *Taenia solium* | PRJNA170813 |
| *Panagrellus redivivus* | PRJNA186477 | *Trichobilharzia regenti* | PRJEB4662 |
| *Parastrongyloides trichosuri* | PRJEB515 |  |  |
| *Pristionchus pacificus* | PRJNA12644 |  |  |
| *Rhabditophanes sp. KR3021* | PRJEB1297 |  |  |
| *Romanomermis culicivorax* | PRJEB1358 |  |  |
| *Soboliphyme baturini* | PRJEB516 |  |  |
| *Steinernema scapterisci* | PRJNA204942 |  |  |
| *Strongyloides ratti* | PRJEB125 |  |  |
| *Strongylus vulgaris* | PRJEB531 |  |  |
| *Syphacia muris* | PRJEB524 |  |  |
| *Teladorsagia circumcincta* | PRJNA72569 |  |  |
| *Thelazia callipaeda* | PRJEB1205 |  |  |
| *Toxocara canis* | PRJEB533 |  |  |
| *Toxocara canis* | PRJNA248777 |  |  |
| *Trichinella spiralis* | PRJNA12603 |  |  |
| *Trichinella spiralis* | PRJNA257433 |  |  |
| *Trichuris suis* | PRJNA179528 |  |  |
| *Trichuris suis* | PRJNA208415 |  |  |
| *Trichuris suis* | PRJNA208416 |  |  |
| *Wuchereria bancrofti* | PRJEB536 |  |  |
| *Wuchereria bancrofti* | PRJNA275548 |  |  |
|  |  |  |  |
| **Seed sequences used for BLAST analysis** | | | |
| **HDM-like** |  | **Sm16-like HDM** |  |
| **Species** | **Accession Number** | **Species** | **Accession Number** |
| *Clonorchis sinensis* | AAM55183 | *Schistosoma haematobium* | KGB37928 |
| *Clonorchis sinensis* | GAA56990 | *Schistosoma haematobium* | KGB37929 |
| *Clonorchis sinensis* | GAA56991 | *Schistosoma japonicum* | CAX74839 |
| *Fasciola hepatica* | CCA61804 | *Schistosoma japonicum* | CAX74840 |
| *Schistosoma haematobium* | XP012797687 | *Schistosoma japonicum* | CAX74838 |
| *Schistosoma japonicum* | CAX69999 | *Schistosoma japonicum* | AAW26100 |
| *Schistosoma japonicum* | CAX69998 | *Schistosoma japonicum* | AAW24692 |
| *Schistosoma japonicum* | CAX69547 | *Schistosoma japonicum* | CAX75220 |
| *Schistosoma japonicum* | CAX70000 | *Schistosoma japonicum* | CAX75221 |
| *Schistosoma mansoni* | CAZ36802 | *Schistosoma japonicum* | CAX75218 |
| *Schistosoma mansoni* | CCD74914 | *Schistosoma japonicum* | AAX30296 |
| *Opisthorchis viverrini* | XP009166563 | *Schistosoma japonicum* | CAX82782 |
| *Opisthorchis viverrini* | XP009166520 | *Schistosoma mansoni* | CAZ38199 |
| *Opisthorchis viverrini* | XP009166519 | *Schistosoma mansoni* | CCD58357 |
| *Opisthorchis viverrini* | XP009166563 |  |  |
