# Supplementary material for: Schistosoma mansoni immunomodulatory molecule Sm16/SPO-1/SmSLP is a member of the trematode-specific helminth defence molecules (HDMs)
Source: PLoS Negl Trop Dis. 2020 Jul 9;14(7):e0008470. doi: 10.1371/journal.pntd.0008470 (PMC7373315; doi:10.1371/journal.pntd.0008470)
Supplement: S3 Table — Numbers represent fold change in cytokine signal. Signal intensity was measured by densitometry. When comparing separate membranes values were normalised using a comparative ratio calculated using densitometry values for membrane positive control spots. (DOCX) [file pntd.0008470.s009.docx]

**S3 Table: Cytokine array analysis of supernatants of THP-1 macrophages that were untreated or treated with Sm16 (34-117), LPS or LPS and Sm16 (34-117).** Numbers represent fold change in cytokine signal. Signal intensity was measured by densitometry. When comparing separate membranes values were normalised using a comparative ratio calculated using densitometry values for membrane positive control spots.

| **Cytokine** | **Untreated v Sm16** | **Untreated v LPS** | **LPS v LPS + Sm16** |
| --- | --- | --- | --- |
| ENA-78 | 1.3 | 0.9 | 0.8 |
| GM-CSF | 26.8 | 44.4 | -0.3 |
| GRO | 4.2 | 1.6 | 1.4 |
| GRO alpha | 2.8 | 3.9 | 0.2 |
| I-309 | 20.4 | 9.9 | 1.3 |
| IL-1 alpha | 1.8 | 0.2 | 1.5 |
| IL-1 beta | 34.4 | 10.1 | 2.1 |
| IL-2 | -0.1 | 0.0 | 2.6 |
| IL-3 | -0.2 | -0.1 | 1.5 |
| IL-4 | 0.3 | -0.1 | 1.6 |
| IL-6 | 309.0 | 703.5 | -0.4 |
| IL-8 | 1.1 | 0.0 | 1.3 |
| IL-10 | 16.9 | 16.3 | 0.5 |
| IL-12 p40/70 | 0.7 | -0.1 | 1.0 |
| IL-15 | 0.9 | -0.1 | 1.3 |
| IFN gamma | 1.4 | -0.4 | 2.4 |
| MCP-1 | 0.0 | 0.6 | 1.1 |
| MCP-2 | 1.5 | 7.5 | 0.2 |
| MCP-3 | 0.6 | 0.9 | 0.5 |
| M-CSF | 0.3 | 0.0 | 1.3 |
| MDC | 3.5 | 2.6 | 0.7 |
| MIP-1 delta | 2.8 | 1.2 | 1.1 |
| RANTES | 1.3 | 0.1 | 1.2 |
| SCF | 1.6 | 0.2 | 1.3 |
| SDF-1 | 0.8 | -0.2 | 1.7 |
| TARC | 0.7 | 0.0 | 1.3 |
| TGF beta 1 | 0.4 | -0.6 | 2.1 |
| TNF alpha | 14.9 | 67.3 | 0.9 |
| TNF beta | 0.5 | 1.1 | 0.5 |
| EGF | 0.5 | 0.4 | 0.7 |
| IGF-1 | 1.0 | 0.2 | 0.8 |
| ANG | 0.6 | -0.3 | 1.6 |
| OSM | 0.6 | -0.1 | 0.8 |
| THPO | 0.5 | -0.2 | 0.8 |
| VEGF | 0.9 | -0.1 | 0.9 |
| PDGF BB | 1.4 | 0.4 | 0.8 |
| Leptin | 0.5 | -0.1 | 0.9 |
