# Supplementary material for: Schistosoma mansoni immunomodulatory molecule Sm16/SPO-1/SmSLP is a member of the trematode-specific helminth defence molecules (HDMs)
Source: PLoS Negl Trop Dis. 2020 Jul 9;14(7):e0008470. doi: 10.1371/journal.pntd.0008470 (PMC7373315; doi:10.1371/journal.pntd.0008470)
Supplement: S4 Table — (DOCX) [file pntd.0008470.s010.docx]

**S4 Table: Top 70 genes differentially regulated by adding Sm16 to THP-1 macrophages.**

| **Down-regulated** | | | **Up-regulated** | | |
| --- | --- | --- | --- | --- | --- |
| *Gene Symbol* | *Fold-Change (Sm16 vs. Untreated)* | *p-value (Sm16 vs. untreated)* | *Gene Symbol* | *Fold-Change (Sm16 vs. untreated)* | *p-value (Sm16 vs. untreated)* |
| **RGS4** | -21.07 | 0.00 | **TNFAIP6** | 41.82 | 0.00 |
| **FLJ14213** | -7.75 | 0.00 | **CXCL2** | 39.24 | 0.00 |
| **LOC157627** | -5.36 | 0.01 | **TNFAIP2** | 31.96 | 0.01 |
| **DLX3** | -5.25 | 0.01 | **CXCL1** | 31.38 | 0.01 |
| **PDK4** | -5.23 | 0.03 | **INDO** | 30.19 | 0.00 |
| **GCNT1** | -5.21 | 0.01 | **CCL4L2** | 29.66 | 0.02 |
| **ZNF280A** | -5.14 | 0.02 | **CSF2** | 24.89 | 0.00 |
| **FAM46C** | -5.14 | 0.00 | **PLAT** | 22.97 | 0.00 |
| **FAM84B** | -4.95 | 0.02 | **LOC728835** | 22.97 | 0.04 |
| **MERTK** | -4.80 | 0.00 | **IL18R1** | 21.86 | 0.00 |
| **ZNF533** | -4.69 | 0.01 | **LAMP3** | 21.33 | 0.00 |
| **HS.12513** | -4.50 | 0.01 | **CCL4L1** | 21.20 | 0.03 |
| **EDNRA** | -4.48 | 0.02 | **TRAF1** | 19.22 | 0.01 |
| **BMP4** | -4.39 | 0.00 | **LOC728830** | 18.09 | 0.01 |
| **C9ORF66** | -3.65 | 0.04 | **IDO1** | 18.04 | 0.00 |
| **HEY2** | -3.59 | 0.01 | **IL1A** | 17.77 | 0.00 |
| **FAM135B** | -3.51 | 0.00 | **IL23A** | 16.98 | 0.00 |
| **SESN1** | -3.47 | 0.02 | **ADORA2A** | 16.39 | 0.00 |
| **LPAR5** | -3.32 | 0.02 | **C2CD4B** | 16.03 | 0.00 |
| **LRMP** | -3.28 | 0.02 | **ABTB2** | 14.06 | 0.01 |
| **TNFAIP8L3** | -3.24 | 0.01 | **TNF** | 14.01 | 0.03 |
| **ZNF385B** | -3.23 | 0.03 | **TMEM166** | 12.43 | 0.01 |
| **KLF4** | -3.21 | 0.02 | **FSTL3** | 12.29 | 0.01 |
| **CD300A** | -3.17 | 0.00 | **VCAM1** | 10.99 | 0.00 |
| **CCR1** | -3.13 | 0.04 | **CD80** | 10.89 | 0.00 |
| **C3ORF54** | -3.09 | 0.01 | **IRAK2** | 10.43 | 0.03 |
| **SPRED1** | -3.07 | 0.00 | **SRC** | 9.94 | 0.01 |
| **KLHDC8B** | -3.04 | 0.01 | **ICAM1** | 9.92 | 0.01 |
| **DAB2** | -3.03 | 0.00 | **CD83** | 9.77 | 0.01 |
| **ST8SIA5** | -2.96 | 0.01 | **CTGF** | 9.65 | 0.01 |
| **RASD2** | -2.94 | 0.00 | **RNF144B** | 9.52 | 0.01 |
| **PKDCC** | -2.92 | 0.01 | **G0S2** | 9.27 | 0.01 |
| **MGC16121** | -2.89 | 0.01 | **IER3** | 9.22 | 0.03 |
| **GFI1** | -2.86 | 0.03 | **STAT4** | 9.07 | 0.00 |
| **ARHGEF3** | -2.84 | 0.04 | **NLF2** | 8.63 | 0.01 |
