# Supplementary material for: Schistosoma mansoni immunomodulatory molecule Sm16/SPO-1/SmSLP is a member of the trematode-specific helminth defence molecules (HDMs)
Source: PLoS Negl Trop Dis. 2020 Jul 9;14(7):e0008470. doi: 10.1371/journal.pntd.0008470 (PMC7373315; doi:10.1371/journal.pntd.0008470)
Supplement: S5 Table — (DOCX) [file pntd.0008470.s011.docx]

**S5 Table: Top 70 genes differentially regulated by adding Sm16 to LPS-treated THP-1 macrophages.**

| **Down-regulated** | | | **Up-regulated** | | |
| --- | --- | --- | --- | --- | --- |
| *Gene Symbol* | *Fold-Change (Sm16 + LPS vs. LPS)* | *p-value (Sm16 + LPS vs. LPS)* | *Gene Symbol* | *Fold-Change (Sm16 + LPS vs. LPS)* | *p-value (Sm16 + LPS vs. LPS)* |
| **LOC728830** | -3.55 | 0.01 | **LOC441763** | 2.60 | 0.00 |
| **CCR7** | -3.29 | 0.01 | **LOC100133565** | 2.59 | 0.01 |
| **IL6** | -3.17 | 0.00 | **EEPD1** | 2.17 | 0.02 |
| **PTGS2** | -3.09 | 0.01 | **LOC100008588** | 2.13 | 0.02 |
| **CCL14** | -2.93 | 0.01 | **C3orf54** | 1.88 | 0.03 |
| **LOC387763** | -2.76 | 0.01 | **MBP** | 1.86 | 0.05 |
| **IL1A** | -2.73 | 0.03 | **AVPI1** | 1.81 | 0.00 |
| **SERPINB2** | -2.65 | 0.04 | **GIMAP6** | 1.78 | 0.02 |
| **MMP10** | -2.46 | 0.05 | **GNPDA1** | 1.74 | 0.02 |
| **IL23A** | -2.46 | 0.04 | **C9orf90** | 1.72 | 0.02 |
| **CSF2** | -2.43 | 0.02 | **ABHD10** | 1.69 | 0.02 |
| **TFPI2** | -2.42 | 0.02 | **HS.534439** | 1.69 | 0.05 |
| **SERPINB7** | -2.35 | 0.01 | **NMRAL1** | 1.68 | 0.02 |
| **GFPT2** | -2.32 | 0.01 | **C15orf39** | 1.67 | 0.01 |
| **CXCL2** | -2.13 | 0.01 | **ST8SIA5** | 1.66 | 0.01 |
| **LOC100130082** | -2.09 | 0.05 | **LBA1** | 1.66 | 0.03 |
| **BCL2A1** | -2.07 | 0.05 | **ID3** | 1.64 | 0.05 |
| **CTGF** | -2.06 | 0.05 | **HSPC047** | 1.63 | 0.02 |
| **LOC644943** | -2.04 | 0.04 | **EPR1** | 1.63 | 0.01 |
| **NKX3-1** | -2.00 | 0.00 | **DAB2** | 1.62 | 0.04 |
| **IL1F9** | -1.97 | 0.02 | **GIMAP4** | 1.59 | 0.02 |
| **SOCS3** | -1.92 | 0.03 | **C20orf177** | 1.59 | 0.04 |
| **SLC7A2** | -1.92 | 0.01 | **PPP1R16B** | 1.58 | 0.03 |
| **HECW2** | -1.90 | 0.01 | **PLEKHH3** | 1.57 | 0.03 |
| **XIRP1** | -1.83 | 0.02 | **ZNF589** | 1.57 | 0.02 |
| **EFNB2** | -1.83 | 0.03 | **NLRX1** | 1.56 | 0.04 |
| **SOCS1** | -1.82 | 0.04 | **CYTH4** | 1.55 | 0.01 |
| **FSTL3** | -1.81 | 0.02 | **SNF8** | 1.55 | 0.04 |
| **CKB** | -1.81 | 0.02 | **C11orf21** | 1.55 | 0.00 |
| **CNKSR3** | -1.80 | 0.01 | **GPT2** | 1.54 | 0.01 |
| **FRMD7** | -1.80 | 0.01 | **CUEDC1** | 1.53 | 0.05 |
| **KDR** | -1.79 | 0.01 | **SH3TC1** | 1.53 | 0.04 |
| **RASL11A** | -1.78 | 0.00 | **TMEM44** | 1.52 | 0.01 |
| **LIPG** | -1.76 | 0.04 | **LOC389286** | 1.52 | 0.02 |
| **IL24** | -1.75 | 0.00 | **SLC9A3R1** | 1.51 | 0.02 |
